# Supplementary material for: An approach to identify gene-environment interactions and reveal new biological insight in complex traits
Source: Nat Commun. 2024 Apr 22;15:3385. doi: 10.1038/s41467-024-47806-3 (PMC11035594; doi:10.1038/s41467-024-47806-3)
Supplement: Supplementary file 1 — Supplementary Information [file 41467_2024_47806_MOESM1_ESM.pdf]

Supplementary Materials for

Xiaofeng Zhu[1#], Yihe Yang[1], Noah Lorincz-Comi[1], Gen Li[1], Amy R. Bentley[2], Paul S de Vries[3], Michael Brown[3], Alanna C. Morrison[3], Charles N. Rotimi[2], W. James Gauderman[4], DC Rao[5], Hugues Aschard[6,7], the CHARGE Gene-lifestyle Interactions Working Group*

Affiliations:
[1]Department of Population and Quantitative Health Sciences, School of Medicine, Case Western Reserve University, Cleveland, Ohio, USA,
[2]Center for Research on Genomics and Global Health, National Human Genome Research Institute, National Institutes of Health, Bethesda, Maryland, USA,
[3]Human Genetics Center, Department of Epidemiology, School of Public Health, The University of Texas Health Science Center at Houston, Houston, Texas, USA,
[4]Division of Biostatistics, Department of Population and Public Health Sciences, University of Southern California, Los Angeles, CA, USA
[5]Center for Biostatistics and Data Science, Institute for Informatics, Data Science and Biostatistics, Washington University School of Medicine, St. Louis, MO, USA
[6]Institut Pasteur, Université Paris Cité, Department of Computational Biology, F-75015 Paris, France
[7]Program in Genetic Epidemiology and Statistical Genetics, Harvard T.H. Chan School of Public Health, Boston, MA 02115, United States
*A list of authors and their affiliations appears at the end of the paper

[#] Corresponding author. Xiaofeng Zhu, Department of Population and Quantitative Health Sciences, Case Western Reserve University, Cleveland, Ohio, USA. E-mail: xxz10@case.edu

Supplementary Note

Statistical modeling: T_{Direct} and $T_{MR\ GxE}$

Case 1: E and G are independent.

1) When GWIS is conducted, we have the following $G \times E$ model to a quantitative trait Y:

$$Y_i = \beta_1 G_i + \beta_2 E_i + \beta_3 (G_i E_i) + \epsilon_i, \qquad \text{(S1)}$$

where β_1, β_2 and β_3 correspond to the main effect of G, the main effect of E and the interaction effect of $G \times E$, respectively, and ϵ_i is a random noise. Without losing generality, we assume

$$E(G_i) = \mu_G,\ g_i = \frac{G_i - \mu_G}{\sigma_G},\ E(E_i) = \mu_E,\ e_i = \frac{E_i - \mu_E}{\sigma_E}.$$

When GWAS is conducted, we have the following model:

$$Y_i = \alpha_0 + \alpha G_i + \varepsilon_i.$$

where α is called the marginal effect. The relationship between marginal effect α, main effect β_1, and interaction effect β_3 is:

$$\alpha = \beta_1 + \mu_E \beta_3, \qquad \text{(S2)}$$

indicating the marginal effect size α is affected by $G \times E$ interactions.

Similar to Aschard (*1*), a standardized version of (S1) is

$$Y_i = \beta_1' g_i + \beta_2' e_i + \beta_3' (g_i e_i) + \epsilon_i', \qquad \text{(S3)}$$

where

$$\beta_1 = \frac{\beta_1'}{\sigma_G} - \frac{\beta_3' \mu_E}{\sigma_E \sigma_G},\ \beta_2 = \frac{\beta_2'}{\sigma_E} - \frac{\beta_3' \mu_G}{\sigma_E \sigma_G},\ \beta_3 = \frac{\beta_3'}{\sigma_E \sigma_G}, \qquad \text{(S4)}$$

Thus

$$\alpha = \beta_1 + \mu_E \beta_3 = \frac{\beta_1'}{\sigma_G}$$

When we perform a linear regression based on standardized regression model (S1), we have

$$\boldsymbol{\beta} = (\boldsymbol{X}^T \boldsymbol{X})^{-1} \boldsymbol{X}^T Y.$$

where $\boldsymbol{X} = [\boldsymbol{1}, \boldsymbol{G}, \boldsymbol{E}, \boldsymbol{GE}]$ is the design matrix, $\Sigma_{\boldsymbol{\beta}} = (\boldsymbol{X}^T \boldsymbol{X})^{-1} \sigma^2$, and σ^2 is the variance of ϵ.

$$\Sigma_{\boldsymbol{\beta}} = \frac{\sigma^2}{n} \begin{bmatrix} E[1] & E[G] & E[E] & E[GE] \\ E[G] & E[G^2] & E[GE] & E[G^2E] \\ E[E] & E[GE] & E[G^2] & E[GE^2] \\ E[GE] & E[G^2E] & E[GE^2] & E[G^2E^2] \end{bmatrix}^{-1}, \qquad \text{(S5)}$$

where n is the sample size in performing GWIS analysis.

When working on g_i and e_i, the standardized of G_i and E_i, it leads

$$\Sigma_{\boldsymbol{\beta}'} = \frac{\sigma^2}{n}\begin{bmatrix} E[1] & 0 & 0 & 0 \\ 0 & E[g^2] & 0 & 0 \\ 0 & 0 & E[e^2] & 0 \\ 0 & 0 & 0 & E[g^2e^2] \end{bmatrix}^{-1} = \frac{1}{n} I_{4\times4}\sigma^2, \tag{S6}$$

where $I_{4\times4}$ is a 4×4 identity matrix. Since we are not interested in the intercept, we will ignore the intercept and let $\boldsymbol{\beta} = [\beta_1, \beta_2, \beta_3]^{\boldsymbol{T}}$. We can calculate the covariance $\Sigma_{\boldsymbol{\beta}}$ through equations (S4), which leads to

$$\Sigma_{\boldsymbol{\beta}} = \frac{\sigma^2}{n\sigma_G^2\sigma_E^2}\begin{bmatrix} \mu_E^2 + \sigma_E^2 & \mu_E\mu_G & -\mu_E \\ \mu_E\mu_G & \mu_G^2 + \sigma_G^2 & -\mu_G \\ -\mu_E & -\mu_G & 1 \end{bmatrix}.$$

Thus, we have

$$var(\alpha) = var(\beta_1 + \mu_E\beta_3) = \frac{\sigma^2}{n\sigma_G^2}, \quad var(\beta_1) = \frac{\sigma^2(\mu_E^2+\sigma_E^2)}{n\sigma_G^2\sigma_E^2},$$
$$var(\beta_2) = \frac{\sigma^2(\mu_G^2+\sigma_G^2)}{n\sigma_G^2\sigma_E^2}, \quad var(\beta_3) = \frac{\sigma^2}{n\sigma_G^2\sigma_E^2}, \quad var(\alpha - \beta_1) = \frac{\mu_E^2\sigma^2}{n\sigma_G^2\sigma_E^2},$$

$$cov(\alpha, \beta_1) = cov(\beta_1 + \mu_E\beta_3, \beta_1) = \frac{\sigma^2}{n\sigma_G^2}, \quad cov(\alpha, \beta_2) = cov(\beta_1 + \mu_E\beta_3, \beta_2) = 0,$$

$$cov(\alpha, \beta_3) = cov(\beta_1 + \mu_E\beta_3, \beta_3) = 0, \quad cov(\alpha - \beta_1, \beta_3) = cov(\mu_E\beta_3, \beta_3) = \frac{\mu_E^2\sigma^2}{n\sigma_G^2\sigma_E^2},$$
$$corr(\alpha - \beta_1, \beta_3) = 1.$$

To test interaction $\beta_3 = 0$, we apply the direct test $T_{Direct} = \frac{\beta_3^2}{var(\beta_3)}$. Alternatively, we can test $\alpha - \beta_1 = 0$ by $T_{diff} = \frac{(\alpha-\beta_1)^2}{var(\alpha-\beta_1)}$. Clearly, T_{Direct} and T_{diff} are the same when G and E are independent and GWAS and GWIS are performed in the same data. T_{Direct} and T_{diff} have the same non-centrality parameter:

$$NCT_{diff} = NCT_{Direct} = \frac{n\sigma_G^2\sigma_E^2\beta_3^2}{\sigma^2}.$$

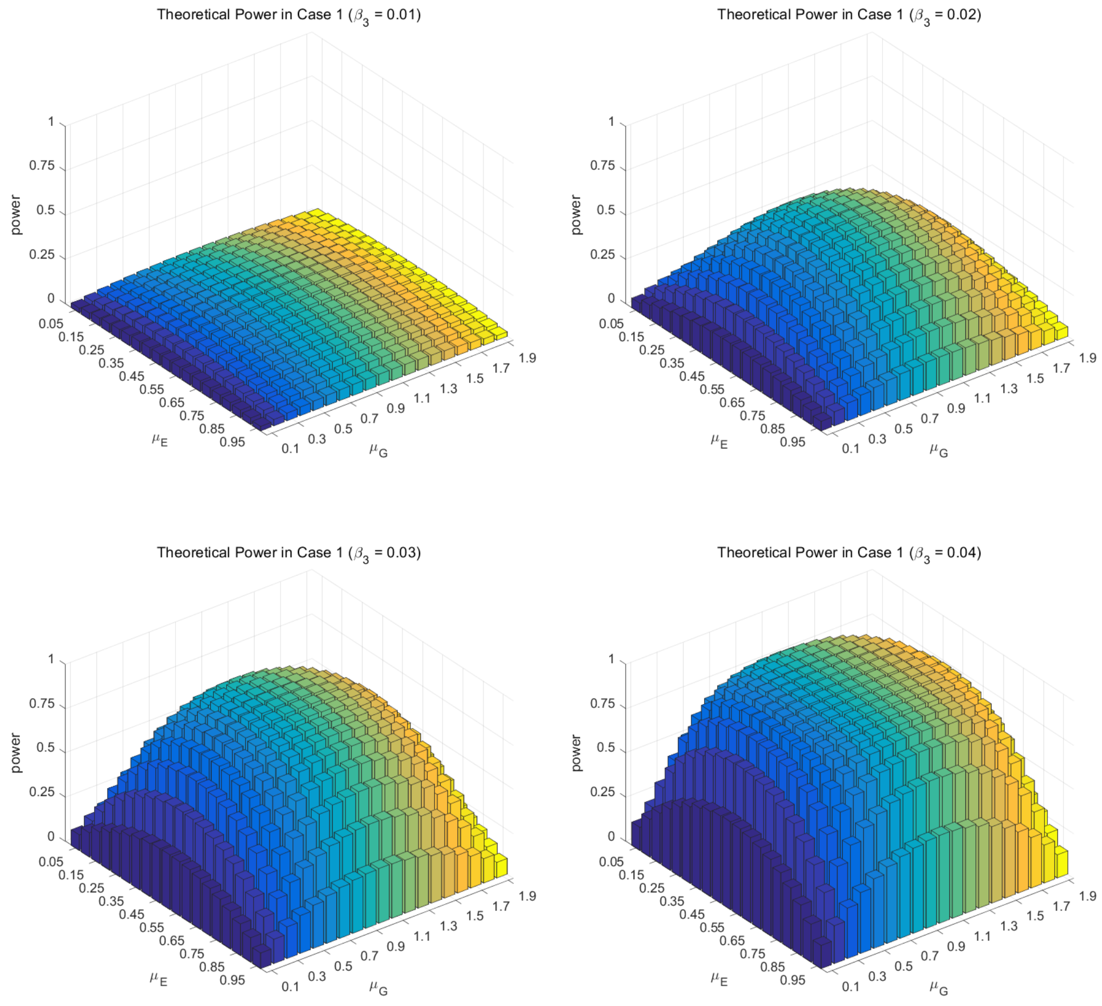

Supplementary Figure 1. The theoretical power of T_{Direct} and T_{diff} under the scenario of Case 1, which is identical. The four subplots correspond to β_3 ranging from 0.01 to 0.04. For each subplot, the x-axis represents the mean μ_G of the variant, the y-axis represents the mean μ_E of the environmental factor, and the z-axis represents the theoretical power. Additionally, the sample size $n = 100K$, and σ was set to be 1.

Case 2: E and G are dependent.

When E and G are correlated, equation (S4) still holds. However, the covariance matrix $\Sigma_{\boldsymbol{\beta}'}$ is

$$\Sigma_{\boldsymbol{\beta}'} = \frac{\sigma^2}{n}\begin{bmatrix} 1 & 0 & 0 & \rho \\ 0 & 1 & \rho & E[g^2 e] \\ 0 & \rho & 1 & E[ge^2] \\ \rho & E[g^2 e] & E[ge^2] & E[g^2 e^2] \end{bmatrix}^{-1}, \tag{S7}$$

where ρ is the correlation between e_i and g_i. To simplify our discussion, we further assume that the environmental factor is mediated by g, which is

$$e_i = \rho g_i + \epsilon_i, \ \ var(\epsilon) = 1 - \rho^2. \tag{S8}$$

Thus,

$$E[g^2 e] = \rho E[g^3] = \rho \frac{1-\mu_G}{\sigma_G}, \tag{S9}$$

$$E[g e^2] = \rho^2 E[g^3] = \rho^2 \frac{1-\mu_G}{\sigma_G}, \tag{S10}$$

$$E[g^2 e^2] = \rho^2 E[g^4] + 1 - \rho^2 = \frac{\rho^2}{\sigma_G^2} + 1 - \rho^2, \tag{S11}$$

and

$$\Sigma_{\boldsymbol{\beta}'} = \frac{\sigma^2}{n}\begin{bmatrix} 1+\rho^2 & \frac{\rho^2(1-\mu_G)}{\sigma_G} & 0 & -\rho \\ \frac{\rho^2(1-\mu_G)}{\sigma_G} & 1 + \frac{\rho^2}{1-\rho^2} + \frac{\rho^2(1-\mu_G)^2}{\sigma_G^2} & \frac{-\rho}{1-\rho^2} & \frac{-\rho(1-\mu_G)}{\sigma_G} \\ 0 & \frac{-\rho}{1-\rho^2} & \frac{1}{1-\rho^2} & 0 \\ -\rho & \frac{-\rho(1-\mu_G)}{\sigma_G} & 0 & 1 \end{bmatrix}. \tag{S12}$$

By using the equations in (S4) and ignoring the intercept, and let $\boldsymbol{\beta} = [\beta_1, \beta_2, \beta_3]^{\boldsymbol{T}}$, we have:

$$\Sigma_{\boldsymbol{\beta}} = \frac{\sigma^2}{n}\begin{bmatrix} \frac{1}{\sigma_G^2}\left[\frac{1}{1-\rho^2} + \left(\frac{\rho(1-\mu_G)}{\sigma_G} + \frac{\mu_E}{\sigma_E}\right)^2\right] & \frac{\rho}{\sigma_G\sigma_E}\left[\frac{-1}{1-\rho^2} + \frac{\mu_G(1-\mu_G)}{\sigma_G^2}\right] + \frac{\mu_G\mu_E}{\sigma_G^2\sigma_E^2} & \frac{-1}{\sigma_G^2\sigma_E}\left[\frac{\rho(1-\mu_G)}{\sigma_G} + \frac{\mu_E}{\sigma_E}\right] \\ \frac{\rho}{\sigma_G\sigma_E}\left[\frac{-1}{1-\rho^2} + \frac{\mu_G(1-\mu_G)}{\sigma_G^2}\right] + \frac{\mu_G\mu_E}{\sigma_G^2\sigma_E^2} & \frac{1}{\sigma_E^2}\left[\frac{1}{1-\rho^2} + \frac{\mu_G^2}{\sigma_G^2}\right] & \frac{-\mu_G}{\sigma_G^2\sigma_E^2} \\ \frac{-1}{\sigma_G^2\sigma_E}\left[\frac{\rho(1-\mu_G)}{\sigma_G} + \frac{\mu_E}{\sigma_E}\right] & \frac{-\mu_G}{\sigma_G^2\sigma_E^2} & \frac{1}{\sigma_G^2\sigma_E^2} \end{bmatrix}, \tag{S13}$$

When G and E are dependent, we have:

$$\alpha = \frac{cov(Y,G)}{\sigma_G^2} = \frac{cov(\beta_1 G + \beta_2 E + \beta_3(GE), G)}{\sigma_G^2}$$

$$= \beta_1 + \frac{\rho\sigma_E}{\sigma_G}\beta_2 + \left(\mu_E + \frac{\rho\sigma_E}{\sigma_G}\right)\beta_3, \tag{S14}$$

which further indicates the marginal effect size α is affected by $G \times E$ interactions and the mediation through E.

By using (S13), we have

$$var(\alpha) = \frac{\sigma^2}{n\sigma_G^2}, \tag{S15}$$

$$cov(\alpha, \beta_3) = cov\left(\beta_1 + \frac{\rho\sigma_E}{\sigma_G}\beta_2 + (\mu_E + \frac{\rho\sigma_E}{\sigma_G})\beta_3, \beta_3\right) = 0, \quad \text{(S16)}$$

$$cov(\alpha, \beta_1) = \frac{\sigma^2}{n\sigma_G^2}, \quad \text{(S17)}$$

$$corr(\alpha, \beta_1) = \frac{1}{\sqrt{\frac{1}{1-\rho^2} + \left(\frac{\rho(1-\mu_G)}{\sigma_G} + \frac{\mu_E}{\sigma_E}\right)^2}}, \quad \text{(S18)}$$

$$var(\alpha - \beta_1) = \frac{\sigma^2}{n\sigma_G^2}\left[\frac{\rho^2}{1-\rho^2} + \left(\frac{\rho(1-\mu_G)}{\sigma_G} + \frac{\mu_E}{\sigma_E}\right)^2\right], \quad \text{(S19)}$$

$$cov(\alpha - \beta_1, \beta_3) = \frac{\sigma^2}{n\sigma_G^2\sigma_E}\left[\frac{\rho(1-\mu_G)}{\sigma_G} + \frac{\mu_E}{\sigma_E}\right], \quad \text{(S20)}$$

$$corr(\alpha - \beta_1, \beta_3) = \frac{\frac{\rho(1-\mu_G)}{\sigma_G} + \frac{\mu_E}{\sigma_E}}{\sqrt{\frac{\rho^2}{1-\rho^2} + (\frac{\rho(1-\mu_G)}{\sigma_G} + \frac{\mu_E}{\sigma_E})^2}}, \quad \text{(S21)}$$

When ρ =0, equation (S21) reduces to $corr(\alpha - \beta_1, \beta_3) = 1$. The direct test $T_{Direct} = \frac{\beta_3^2}{var(\beta_3)}$ still tests the $G \times E$ interaction, but testing $\alpha - \beta_1 = 0$ by $T_{diff} = \frac{(\alpha-\beta_1)^2}{var(\alpha-\beta_1)}$ tests for $\frac{\rho\sigma_E}{\sigma_G}\beta_2 + \left(\mu_E + \frac{\rho\sigma_E}{\sigma_G}\right)\beta_3 = 0$, which is testing for the combination of mediation (ρ) and interaction (β_3).

Above discussion suggests that when mediation is present, T_{diff} will also detect mediation even there is no $G \times E$ interaction. However, we can test the $G \times E$ interaction through two step procedure: 1) We apply T_{diff} to search for variants with joint effect of mediation and interaction effect; 2) we apply T_{Direct} for the variants detected by T_{diff}. Although T_{diff} and T_{Direct} are correlated, this procedure seems to have a good control of the type I error rate in the simulations which mimic the real data. The reason is that we can exclude the genetic variants strongly associated with the environment factor from the GWAS of E. Therefore, the contribution of mediation has little effect (see the simulation results Fig 3 and Fig S6). It also improves the power because of the mediation and the reduction of multiple test burden.

When mediation effect presents, T_{diff} has a non-centrality parameter:

$$NCT_{diff} = \frac{n\sigma_G^2\left[\frac{\rho\sigma_E}{\sigma_G}(\beta_2 + \beta_3) + \mu_E\beta_3\right]^2}{\sigma^2[\frac{\rho^2}{1-\rho^2} + (\frac{\rho(1-\mu_G)}{\sigma_G} + \frac{\mu_E}{\sigma_E})^2]}, \qquad NCT_{Direct} = \frac{n\sigma_G^2\sigma_E^2\beta_3^2}{\sigma^2}$$

when $\rho \neq 0$, T_{diff} can be more powerful than T_{Direct}.

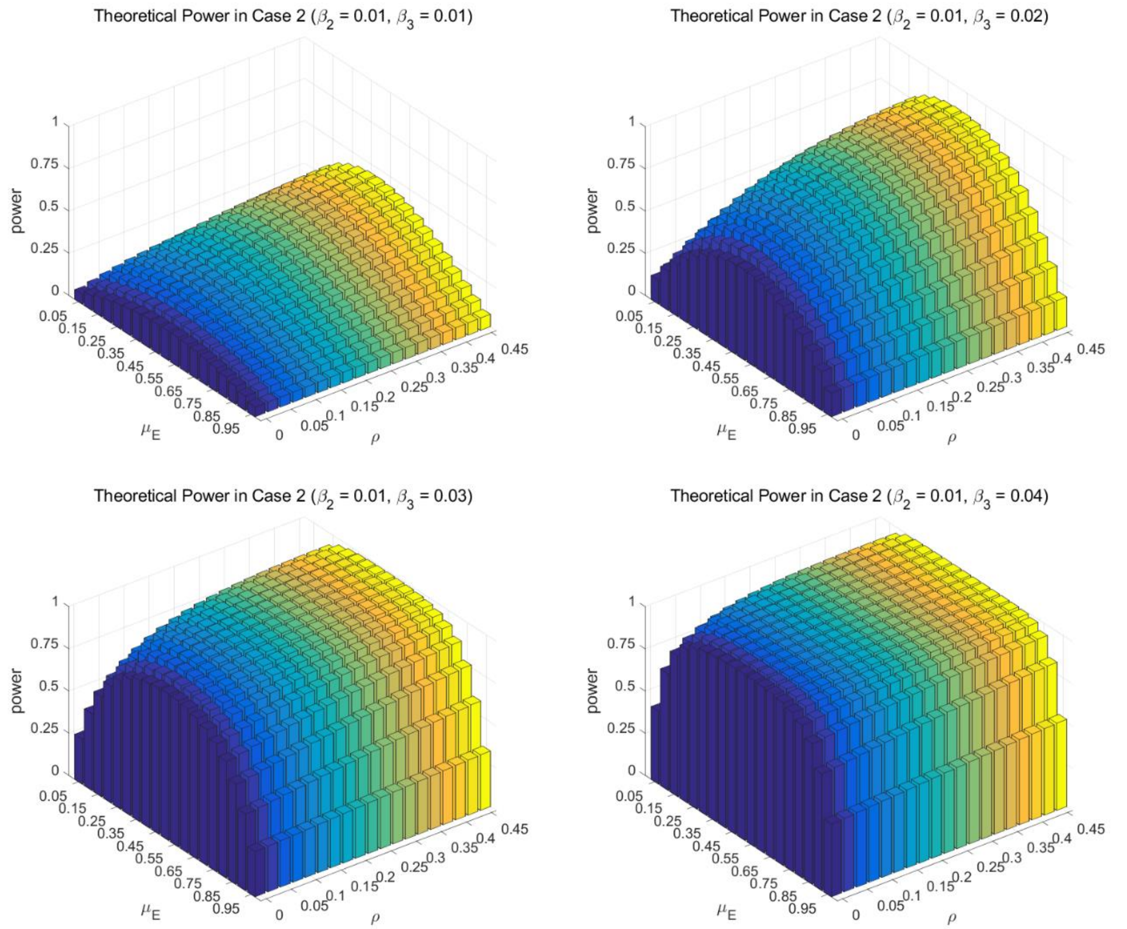

Supplementary Figure 2. The theoretical power of T_{diff} under the scenario of Case 2. For each subplot, the x-axis represents the correlation coefficient ρ of mediation, the y-axis represents the mean μ_E of the environmental factor, and the z-axis represents the theoretical power. Additionally, the sample size is $n = 100K$, the genotype has allele frequency or mean μ_G=0.6. σ was set to 1. As the correlation coefficient ρ of mediation increases, the power of T_{diff} significantly rises. Furthermore, due to the variance changes in NCT_{diff}, the power does not symmetrically decrease as μ_E increases or decreases from 0.5. In fact, the decline in power with an increase in μ_E is slightly faster than the decrease in power with a reduction in μ_E from 0.1.

Case 3. GWAS and GWIS are performed in different samples with overlapping.

We are now working on the case when GWAS and GWIS are performed in different sample sizes. Let n_1, n_2 and n_0 represent the sample sizes of GWAS, GWIS and overlapping sample between GWAS and GWIS. In this case, the marginal effect α is estimated from GWAS and $\beta_1, \beta_2, \beta_3$ are estimated from GWIS, respectively. We can deduce the covariance between α and $\beta_1, \beta_2, \beta_3$ using the work in Case 1 and 2.

Let a random variable I takes 1 when samples are overlapped and 0 when samples are not overlapped. The estimates of α and $\boldsymbol{\beta}$ are the weighted estimates of them in overlapped sample and non-overlapped samples. That is,

$$\alpha = \alpha^{(O)}P(I=1) + \alpha^{(n_1\backslash O)}P(I=0),$$

$$\boldsymbol{\beta} = \boldsymbol{\beta}^{(O)}P(I=1) + \boldsymbol{\beta}^{(n_2\backslash O)}P(I=0),$$

where index (O), $(n_1\backslash O)$ and $(n_2\backslash O)$ represent overlapped, GWAS samples after excluding the overlapped, and GWIS samples after excluding the overlapped samples, which lead to the following:

$$\alpha = \frac{n_o}{n_1}\alpha^{(O)} + \frac{n_1 - n_o}{n_1}\alpha^{(n_1 O)},$$

$$\boldsymbol{\beta} = \frac{n_o}{n_2}\boldsymbol{\beta}^{(O)} + \frac{n_2 - n_o}{n_2}\boldsymbol{\beta}^{(n_2\backslash O)},$$

$$cov(\alpha - \beta_1, \beta_3) = cov\left(\frac{n_o}{n_1}\alpha^{(O)} + \frac{n_1 - n_o}{n_1}\alpha^{(n_1 O)}, \frac{n_o}{n_2}\beta_3^{(O)} + \frac{n_2 - n_o}{n_2}\beta_3^{(n_1\backslash O)}\right) - cov(\beta_1, \beta_3)$$
$$= cov\left(\frac{n_o}{n_1}\alpha^{(O)}, \frac{n_o}{n_2}\beta_3^{(O)}\right) - cov(\beta_1, \beta_3) = -cov(\beta_1, \beta_3)$$
$$= \frac{\sigma^2}{n_2\sigma_{G2}^2\sigma_{E2}}\left[\frac{\rho(1-\mu_{G2})}{\sigma_{G2}} + \frac{\mu_{E2}}{\sigma_{E2}}\right], \qquad \text{(S22)}$$

Thus, $cov(\alpha, \beta_3) = cov(\alpha - \beta_1, \beta_3) + cov(\beta_1, \beta_3) = 0$

$$cov(\alpha, \beta_1) = cov\left(\frac{n_o}{n_1}\alpha^{(O)} + \frac{n_1 - n_o}{n_1}\alpha^{(n_1 O)}, \frac{n_o}{n_2}\beta_1^{(O)} + \frac{n_2 - n_o}{n_2}\beta_1^{(n_1\backslash O)}\right)$$
$$= \frac{n_o\sigma^2}{n_1 n_2 \sigma_{G0}^2}, \qquad \text{(S23)}$$

$$corr(\alpha, \beta_1) = \frac{n_o\sigma_{G1}\sigma_{G2}}{\sigma_{G0}^2\sqrt{n_1 n_2[\frac{1}{1-\rho^2} + \left(\frac{\rho(1-\mu_{G2})}{\sigma_{G2}} + \frac{\mu_{E2}}{\sigma_{E2}}\right)^2]}}, \qquad \text{(S24)}$$

where μ_{G2}, σ_{G2}, μ_{E2}, and σ_{E2} refer to the mean and standard deviation of G and E in GWIS samples, μ_{G1}, σ_{G1}, μ_{E1}, and σ_{E1} refer to the mean and standard deviation of G and E in GWAS samples, and μ_{G0}, σ_{G0}, μ_{E0}, and σ_{E0} refer to the mean and standard deviation of G and E in the overlapped samples by GWAS and GWIS, respectively. Then we have the following:

$$\alpha - \beta_1 = \frac{\rho\sigma_{E1}}{\sigma_{G1}}\beta_2 + \left(\mu_{E1} + \frac{\rho\sigma_{E1}}{\sigma_{G1}}\right)\beta_3$$

and

$$var(\alpha - \beta_1) = \sigma^2\left[\frac{1}{n_1\sigma_{G1}^2} - \frac{2n_0}{n_1 n_2\sigma_{G0}^2} + \frac{1}{n_2\sigma_{G2}^2}\left(\frac{1}{1-\rho^2} + \left(\frac{\rho(1-\mu_{G2)}}{\sigma_{G2}} + \frac{\mu_{E2}}{\sigma_{E2}}\right)^2\right)\right]. \qquad \text{(S25)}$$

T_{diff} has a non-centrality parameter:

$$NCT_{diff} = \frac{(\frac{\rho\sigma_{E1}}{\sigma_{G1}}\beta_2 + \left(\mu_{E1} + \frac{\rho\sigma_{E1}}{\sigma_{G1}}\right)\beta_3)^2}{\sigma^2[\frac{1}{n_1\sigma_{G1}^2} - \frac{2n_0}{n_1n_2\sigma_{G0}^2} + \frac{1}{n_2\sigma_{G2}^2}(\frac{1}{1-\rho^2} + \left(\frac{\rho(1-\mu_{G2})}{\sigma_{G2}} + \frac{\mu_{E2}}{\sigma_{E2}}\right)^2)]}$$

and T_{Direct} has a non-centrality parameter:

$$NCT_{Direct} = \frac{n_2\sigma_{G2}^2\sigma_{E2}^2\beta_3^2}{\sigma^2}.$$

Noted that $cov(\alpha, \beta_3) = 0$ is also hold when β_3 is estimated from the GWAS sample excluding GWIS sample. As a result, T_{diff} is independent of β_3 when the β_3 is estimated from the GWAS sample excluding GWIS sample. Therefore, the direct test T_{Direct} using the GWAS sample after excluding GWIS sample is an independent replication for either T_{diff} or GWIS T_{Direct} test.

T_{diff} is still testing for the combined effect of mediation and interaction and its power depends on the environmental mean and variance in the GWAS data. Again, we can test the $G \times E$ interaction through the two-step procedure: 1) We apply T_{diff} to search variants with joint effect of mediation and interaction effect; 2) we apply T_{Direct} for the variants detected by T_{diff}. Since GWAS is often conducted in much larger sample size than GWIS, the power of T_{diff} is increased, therefore, the two-step procedure for testing interactions is also increased.

In practice, the GWIS sample is often a subset of GWAS. In this case, $n_0 = n_2$, which leads to

$$NCT_{diff} = \frac{(\frac{\rho\sigma_{E1}}{\sigma_{G1}}\beta_2 + \left(\mu_{E1} + \frac{\rho\sigma_{E1}}{\sigma_{G1}}\right)\beta_3)^2}{\sigma^2[\frac{1}{n_1\sigma_{G1}^2} - \frac{2}{n_1\sigma_{G2}^2} + \frac{1}{n_2\sigma_{G2}^2}(\frac{1}{1-\rho^2} + \left(\frac{\rho(1-\mu_{G2})}{\sigma_{G2}} + \frac{\mu_{E2}}{\sigma_{E2}}\right)^2)]}.$$

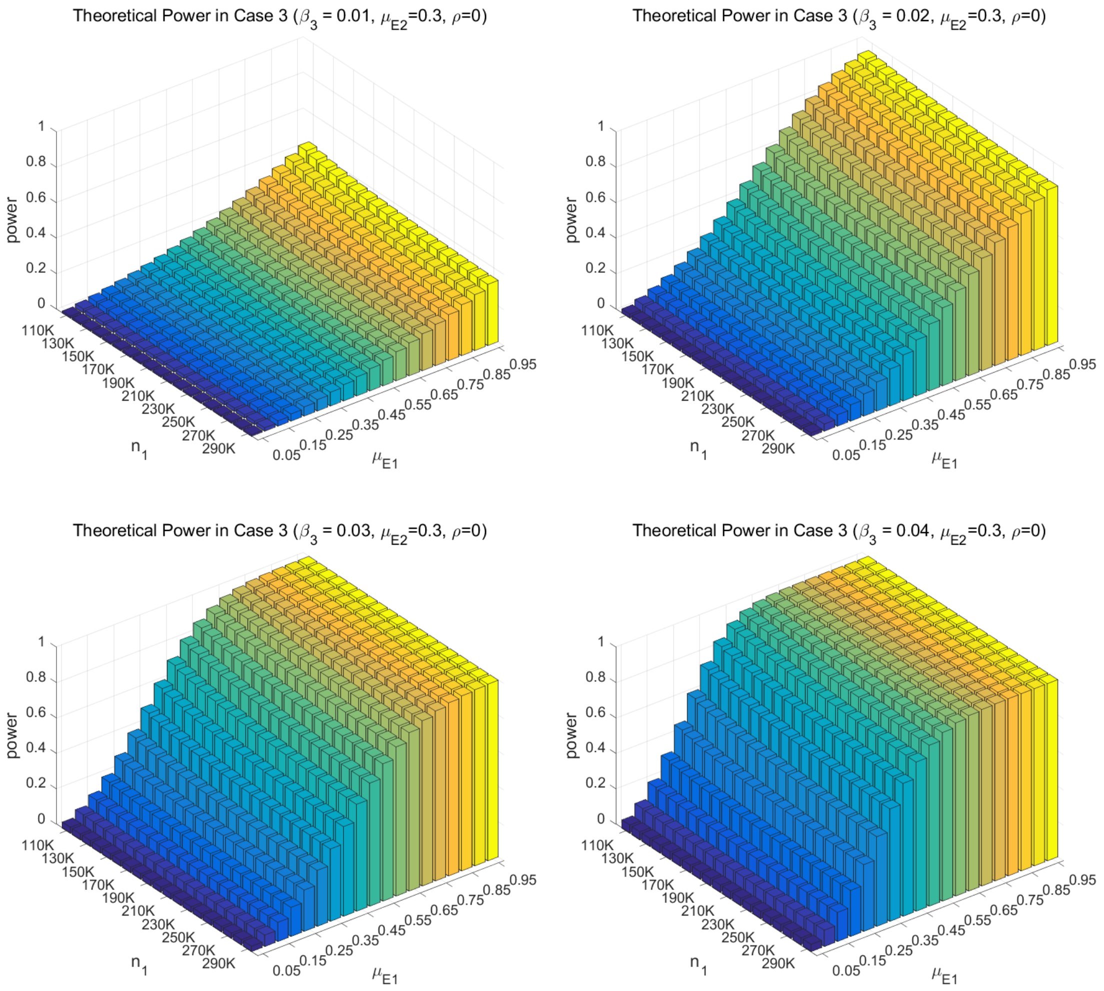

Supplementary Figure 3. The theoretical power of T_{diff} under the scenario of Case 3. For each subplot, the x-axis represents the mean μ_{E1} of the environmental factor, the y-axis represents the sample size n_1 in the GWAS data, and the z-axis represents the theoretical power. Additionally, the sample size in the GWIS data is $n_2 = 100K$, the mean μ_{E2} of the environmental factor in the GWIS data is 0.3, the mean μ_G of the variant is 0.6, the correlation coefficient of mediation $\rho = 0$, and the variance of the random error $\sigma = 1$. It can be observed that as μ_{E1} increases relative to the data in the GWIS, the power substantially rises and is insensitive to the increase in n_1.

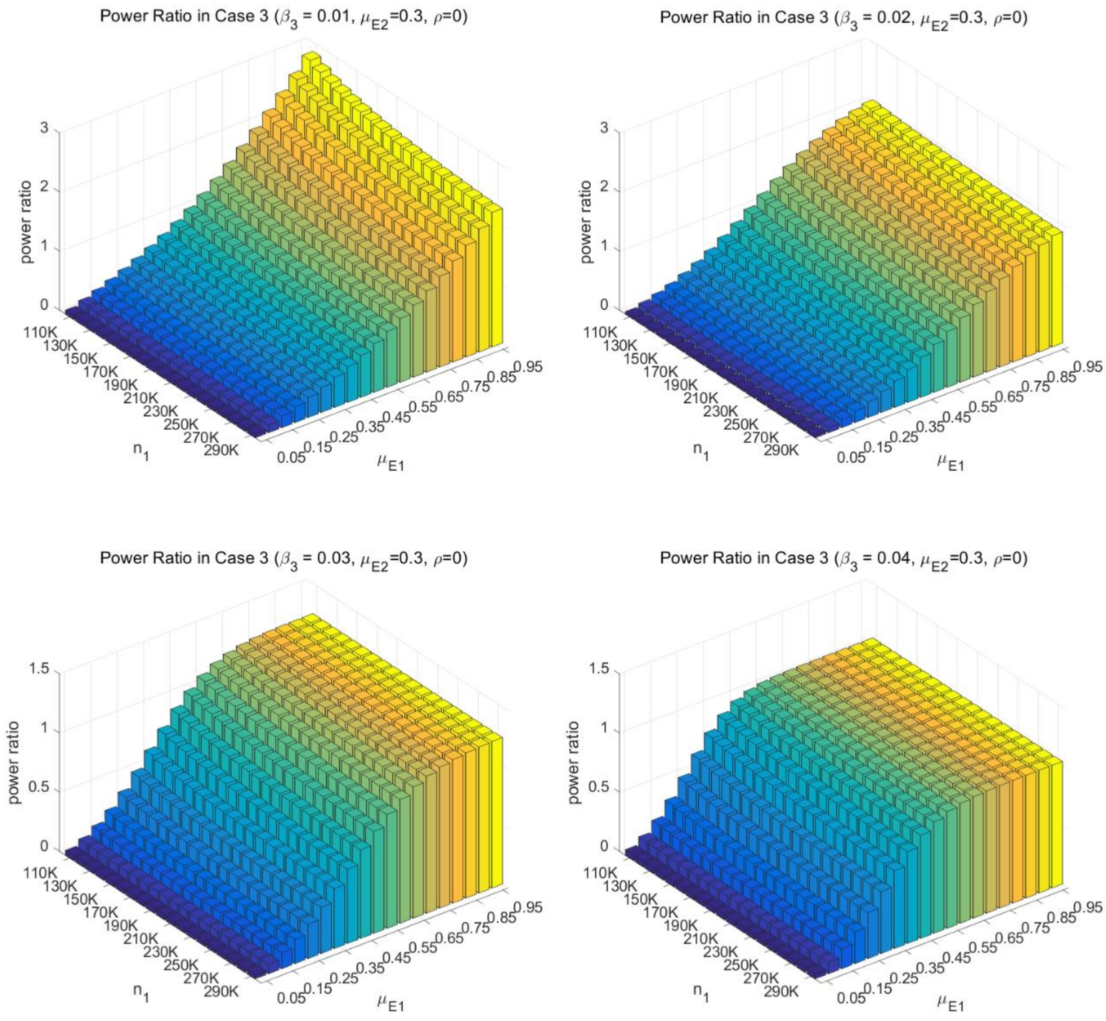

Supplementary Figure 4. The comparison between T_{diff} and T_{direct} under the scenario of Case 3 but no mediation. The z-axis represents the ratio of the power of T_{diff} over the power of T_{direct}. If this ratio is larger than 1, then T_{diff} is more powerful than than T_{direct}. Generally, T_{diff} is more powerful than T_{direct} if $\mu_{E2} > 0.5$ and $\mu_{E1} = 0.3$.

Supplementary Figure 5. The scatterplots between z-scores of T_{MR_GxE} test based on $\left(\hat{\alpha} - \hat{\theta}\beta_1\right)$ and direct test (T_{Direct}) based on effect sizes $\hat{\beta}_3$ obtained in GLI data. Because the GWAS was performed with smoking status as a covariate, the z-scores of T_{MR_GxE} and T_{Direct} should be similar. The Pearson correlation coefficient between z-scores of T_{MR_GxE} test and T_{Direct} is 0.977, which is consistent. The red straight line represents the regression line.

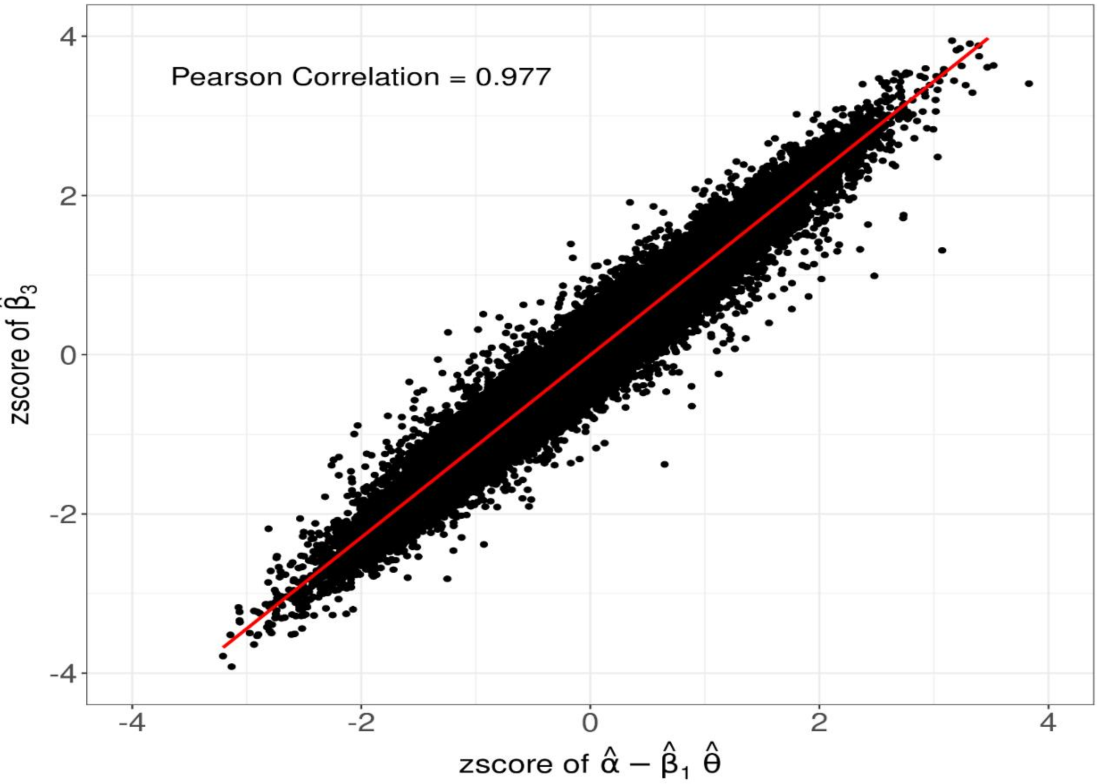

Supplementary Figure 6. The estimations of θ, interaction effect β_3, and the comparison of type I error and power for T_{MR_GXE} and the direct test for the $G \times E$ interaction when there is no mediation. The GWAS sample size is two times of GWIS sample size and GWIS sample is the subset of GWAS sample: $n_1 = 2n_2 = 2n_0$. The sample size n_2 increases from 60k to 150k. We set $\mu_E^{mar} = 1$ and $\mu_E^{int} = 0.5$. (a). Box plots of $\hat{\theta}$ in simulations under different GWIS sample sizes. The top and bottom edges of the box plots represent the 25th and 75th percentiles of $\hat{\theta}$, and the horizontal middle line represents the 50th percentile. The vertical bars extend from the 25th (or 75th) percentile of $\hat{\theta}$ to the minimum (or maximum) value of simulated data. $E(\hat{\theta})$ converges to 1 as sample size increases. (b). Box plots of the direct estimate of β_3 in GWIS (top panel) and by $(\hat{\alpha} - \hat{\beta}_1\hat{\theta})/\mu_e$ through MR- $G \times E$ analysis (bottom panel). The box plots are interpreted the same as in **A** accordingly. The direct estimate of β_3 in GWIS or by $(\hat{\alpha} - \hat{\beta}_1\hat{\theta})/\mu_e$ through MR-GxE analysis are both unbiased. Here s=-1 refers to the main effect and interaction effect have opposite effect directions; s=0 refers no main effect; and s=1 refers that the main effect and interaction effect have the same effect direction. (c). Type I error rate comparison between T_{MR_GxE} and the direct test for different main and interaction effect directions. Both T_{MR_GxE} and the direct test maintain the type I error well. (d) Power comparison between T_{MR_GxE} and the direct test for different main and interaction effect directions.

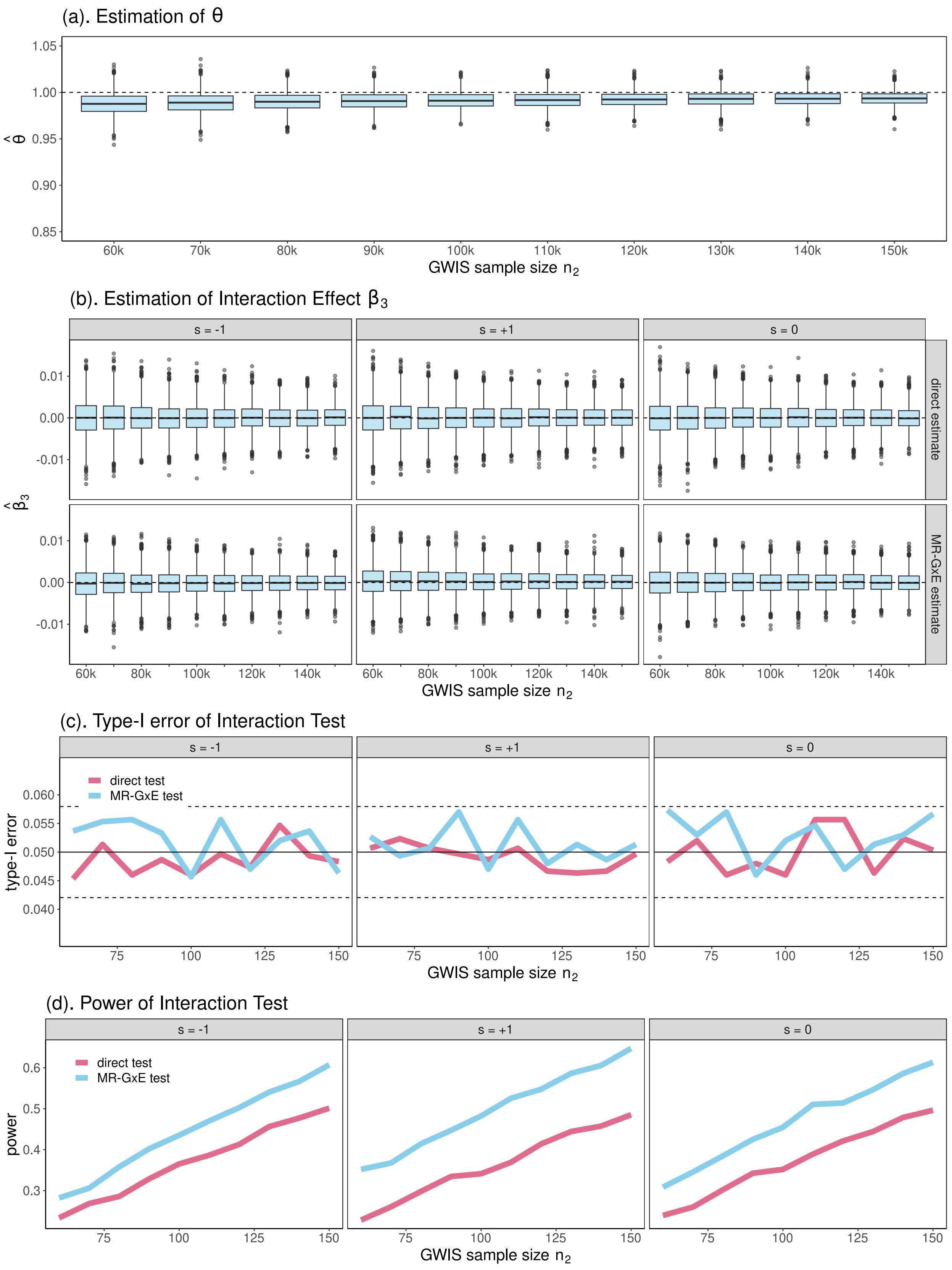

(a). Estimation of θ
GWIS sample size n_2
60k
70k
80k
90k
100k
110k
120k
130k
140k
150k
$\hat{\theta}$
1.05
1.00
0.95
0.90
0.85
(b). Estimation of Interaction Effect β_3
s = -1
s = +1
s = 0
direct estimate
MR-GxE estimate
$\hat{\beta}_3$
0.01
0.00
-0.01
60k
80k
100k
120k
140k
GWIS sample size n_2
(c). Type-I error of Interaction Test
direct test
MR-GxE test
type-I error
0.060
0.055
0.050
0.045
0.040
75
100
125
150
GWIS sample size n_2
(d). Power of Interaction Test
power
0.6
0.5
0.4
0.3
GWIS sample size n_2

Supplementary Figure 7. (a) and (c): direct estimate $\hat{\beta}_3$ and MR-GxE estimate $\left(\hat{\alpha} - \hat{\beta}_1\hat{\theta}\right)/\mu_E^{mar}$ of interaction effect when true $\beta_3 = 0$. (b) and (d): direct estimate $\hat{\beta}_3$ and MR-GxE estimate $\left(\hat{\alpha} - \hat{\beta}_1\hat{\theta}\right)/\mu_E^{mar}$ of interaction effect when true $\beta_3 = 0.005$. Settings of (a) and (b): $\mu_E^{mar} = 1$, $\mu_E^{int} = 0.5$, $n_1 = 2n_2 = 2n_0$ where n_2 increases from 60k to 150k. Settings of (c) and (d): $n_1 = 160k$, $n_2 = 80k$, $n_0 = 80k$, μ_E^{int} is fixed to 0.5, μ_E^{mar} increases from 0.1 to 1.5, and environment factor mean ratio increases from 0.2 to 3. In each panel. the top and bottom edges of the box plots represent the 25th and 75th percentiles of the estimate, and the horizontal middle line represents the 50th percentile. The vertical bars extend from the 25th (or 75th) percentile of $\hat{\theta}$ to the minimum (or maximum) value of simulated data. The simulation results suggest that the direct estimate $\hat{\beta}_3$ and MR-GxE estimate $\left(\hat{\alpha} - \hat{\beta}_1\hat{\theta}\right)/\mu_E^{mar}$ are all unbiased.

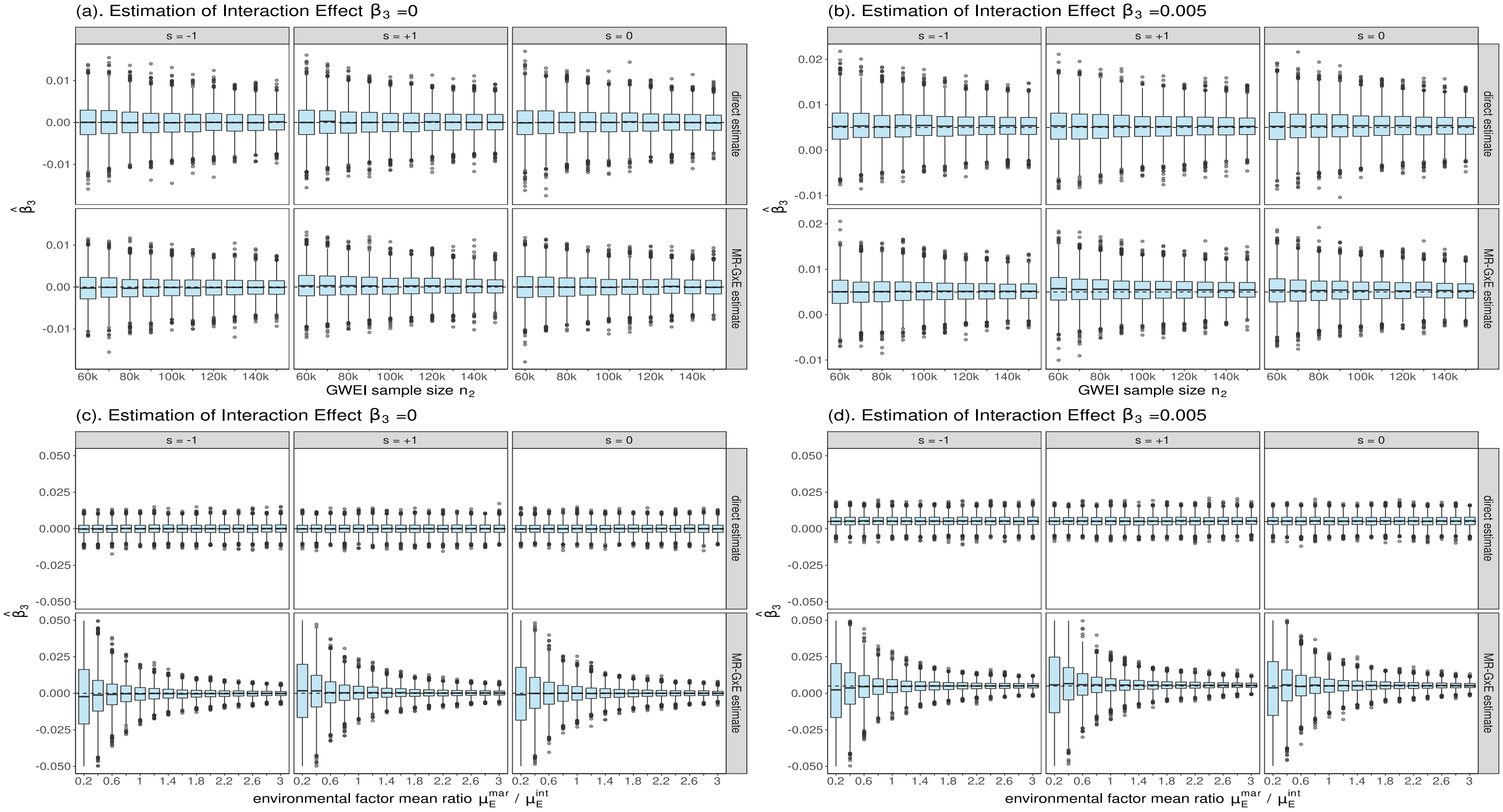

Supplementary Figure 8. The estimates of θ and β_3 when GWAS and GWIS are performed in different samples or the same samples. (a): estimate of θ, left: there is no sample overlapping between GWAS and GWIS; right: GWAS and GWIS were performed in the sample. (b): direct estimate $\hat{\beta}_3$ and MR-GxE estimate $\left(\hat{\alpha} - \hat{\beta}_1\hat{\theta}\right)/\mu_E^{mar}$ of interaction effect. Settings: $n_1 = 200k$, $n_2 = 80k$, $n_0 = 0$ (0% sample overlap) or $n_0 = 80k$ (100% sample overlap), $\mu_E^{int} = 0.5$, μ_E^{mar} increases from 0.32 to 2, and environment factor mean ratio increases from 0.64 to 4. In each panel. the top and bottom edges of the box plots represent the 25th and 75th percentiles of the estimate, and the horizontal middle line represents the 50th percentile. The vertical bars extend from the 25th (or 75th) percentile of $\hat{\theta}$ to the minimum (or maximum) value of simulated data.

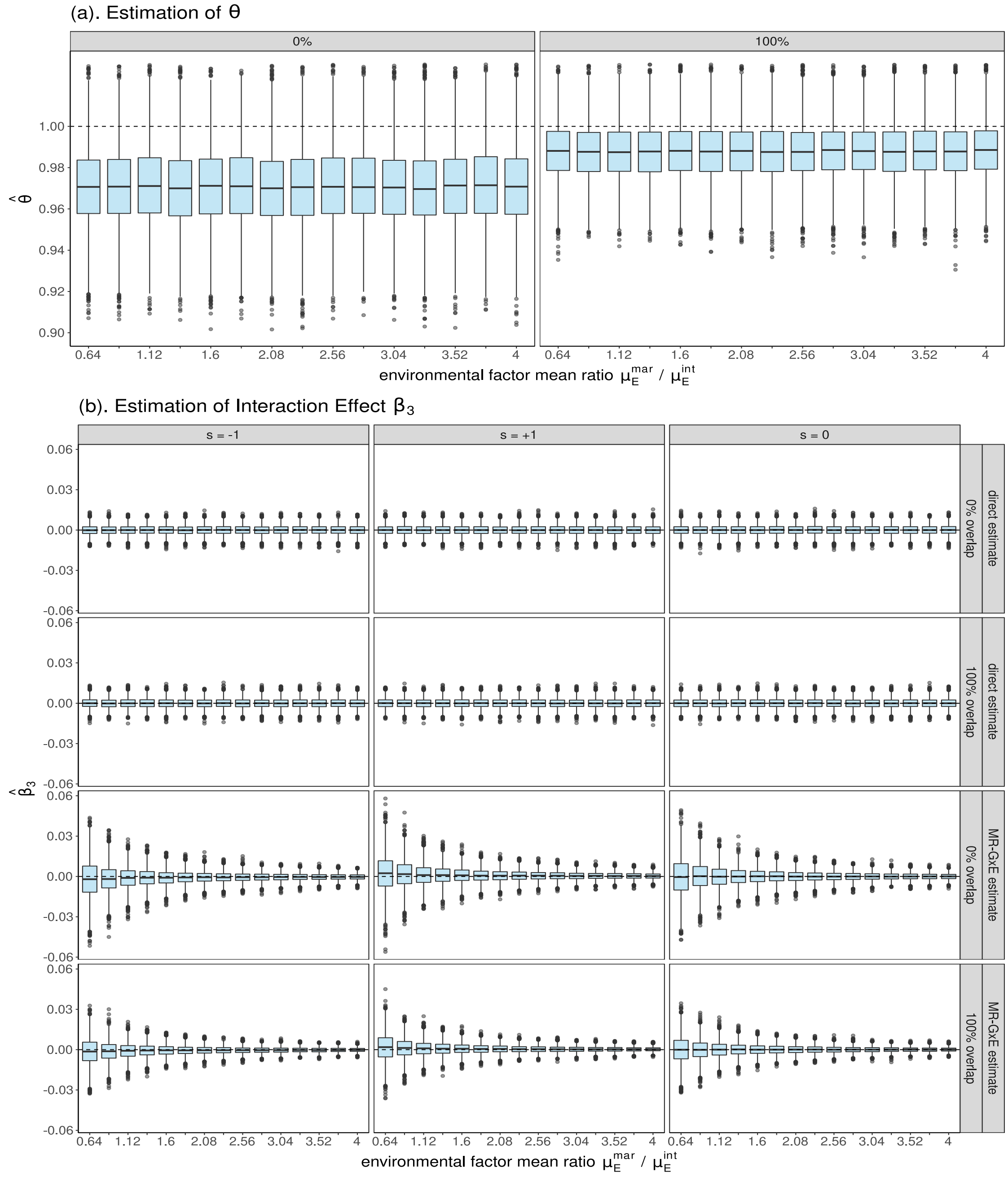

Supplementary Figure 9. (a): Type-I errors of direct test T_{direct} and MR-GxE test T_{MR-GxE}. The dash lines represent the 95% CI. b) Power of direct test T_{direct} and MR-GxE test T_{MR-GxE}. Settings: $n_1 = 200k$, $n_2 = 80k$, $n_0 = 0$ (0% sample overlap) or $n_0 = 80k$ (100% sample overlap), $\mu_E^{int} = 0.5$, μ_E^{mar} increases from 0.32 to 2, and environment factor mean ratio increases from 0.64 to 4.

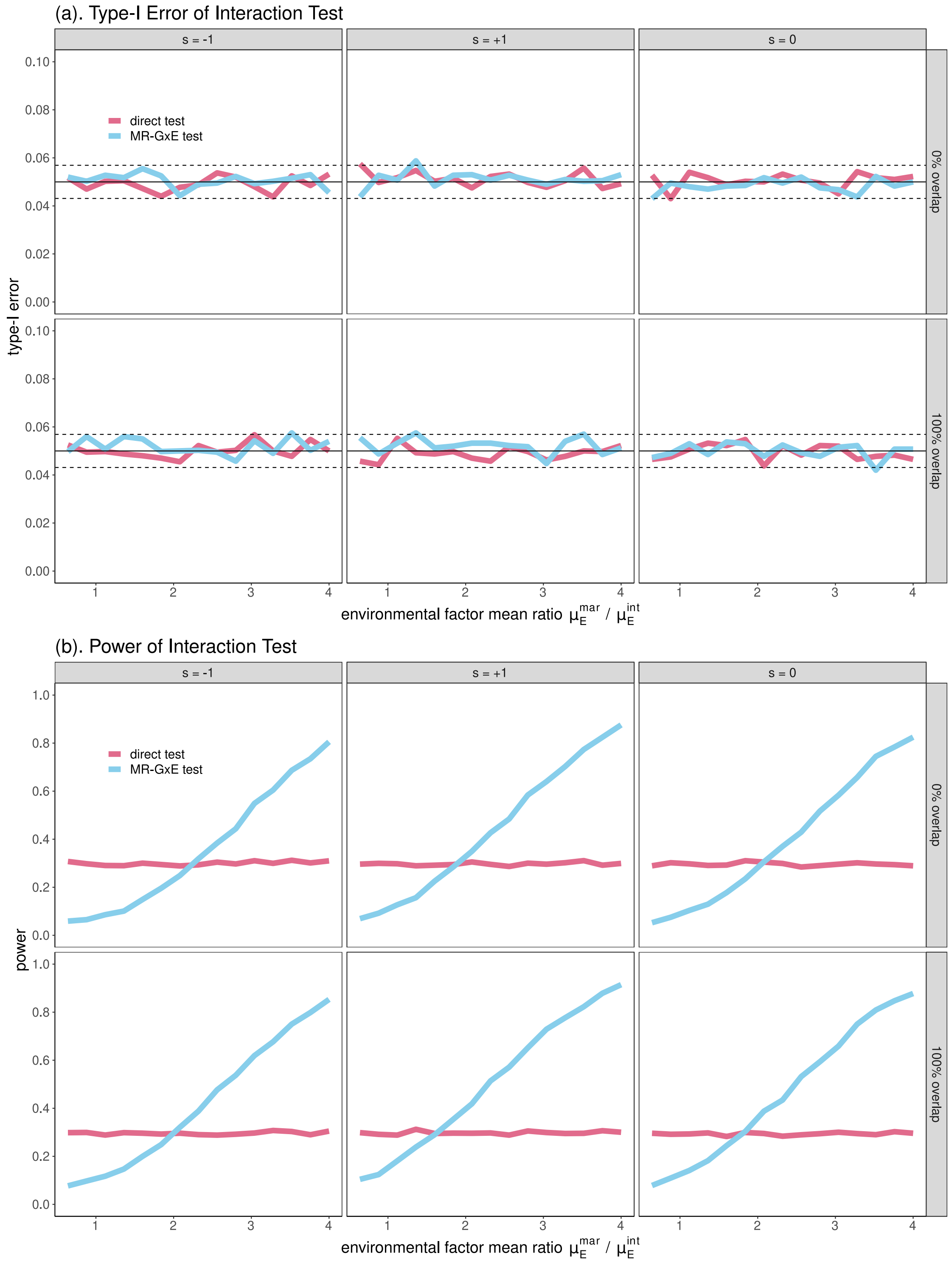

Supplementary Figure 10. Type I error and power for T_{Direct} (red), T_{MR_GxE} (green) and two-step (blue). In brief, we simulated a continuous trait, environmental factor and 20 independent genetic variants for 1000 times. The type I error and power for T_{Direct} and T_{MR_GxE} were calculate by correcting for 20 tests using the Bonferroni correction. The environment mean was set to 0.5. For the two-step procedure, we first applied T_{MR_GxE} and Bonferroni correction. The variants survived after T_{MR_GxE} were further tested by T_{Direct} and Bonferroni correction was also applied. The sample size for marginal effect estimation varied from $n_2 = 20{,}000\ to\ 300{,}000$. The sample size for the main effect estimate was fixed to $n_2 = 20{,}000$. **A**. All the variants have no contribution of either mediation or GxE interaction. **B**. One variant has mediation effect and accounts for 0.25% of environment variation, and E contributes 1% of phenotypic variation. **C**. One variant has mediation effect and accounts for 0.25% of environment variation, and E contributes 5% of phenotypic variation. The dash line represents the 5% typer I error rate. **D**. One variant has GxE interaction but no mediation. **E**. One variant has both mediation and GxE interaction. This variant accounts for 0.25% of environment variation, and E contributes 1% of phenotypic variation. **F**. One variant has both mediation and GxE interaction. This variant accounts for 0.25% of environment variation, and E contributes 5% of phenotypic variation. The simulations suggested the type I error rate is in general well controlled except when E has a large contribution to the phenotype when GWAS and GWIS are performed in the same dataset. Mediation effect improves the power to detect GxE for T_{Direct}, T_{MR_GxE} and two-step, with more

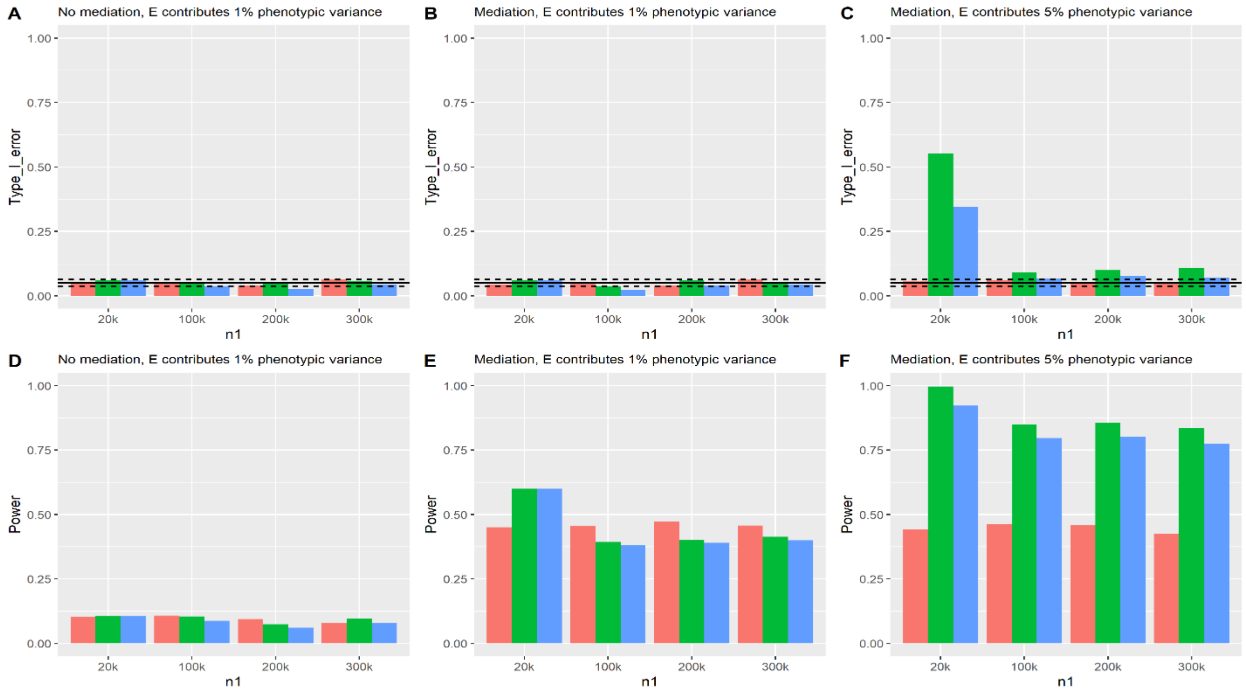

Supplementary Figure 11.1. Zoomed locus-specific plots for the GxE loci identified by T_{MR-GxE} for LDL-C. In each panel, top is -log10(P-value) and bottom is the corresponding CADD and RegulomDB score generated by software FUMA. (a) *APOE/BCAM* , Current Drinking. (b) *SUGP1*, Current Drinking. (c) *SMARCA4*, Current drinking. (d) *APOB*, regular drinking. (e) *SMARCA4*, Regular drinking. (f) *SUGP1*, Regular Drinking. (g) *APOE/BCAM* , Regular Drinking.

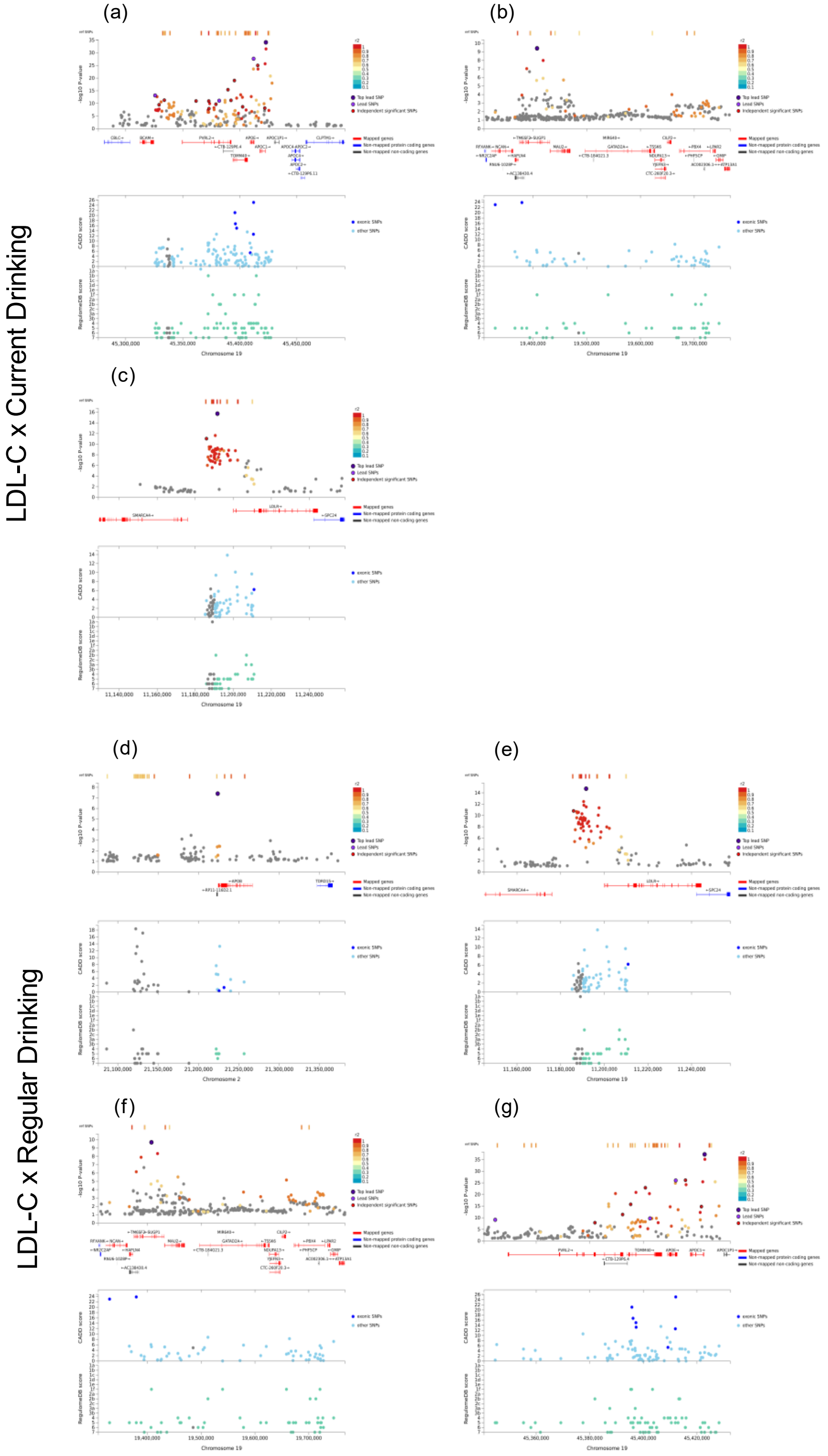

Supplementary Figure 11.2. Zoomed locus-specific plots for the GxE loci identified by T_{MR-GxE} for LDL-C. In each panel, top is -log10(P-value) and bottom is the corresponding CADD and RegulomDB score generated by software FUMA. (a) *SMARCA4*, Current Smoking. (b) *SUGP1*, Current Smoking. (c) AC008897.2, Current smoking. (d) *APOE*, current smoking. (e) *CELSR2/PSRC1*, Ever Smoking. (f) *APOE/BCAM* , Ever Smoking. (g) *SMARCA4*, Ever Smoking.

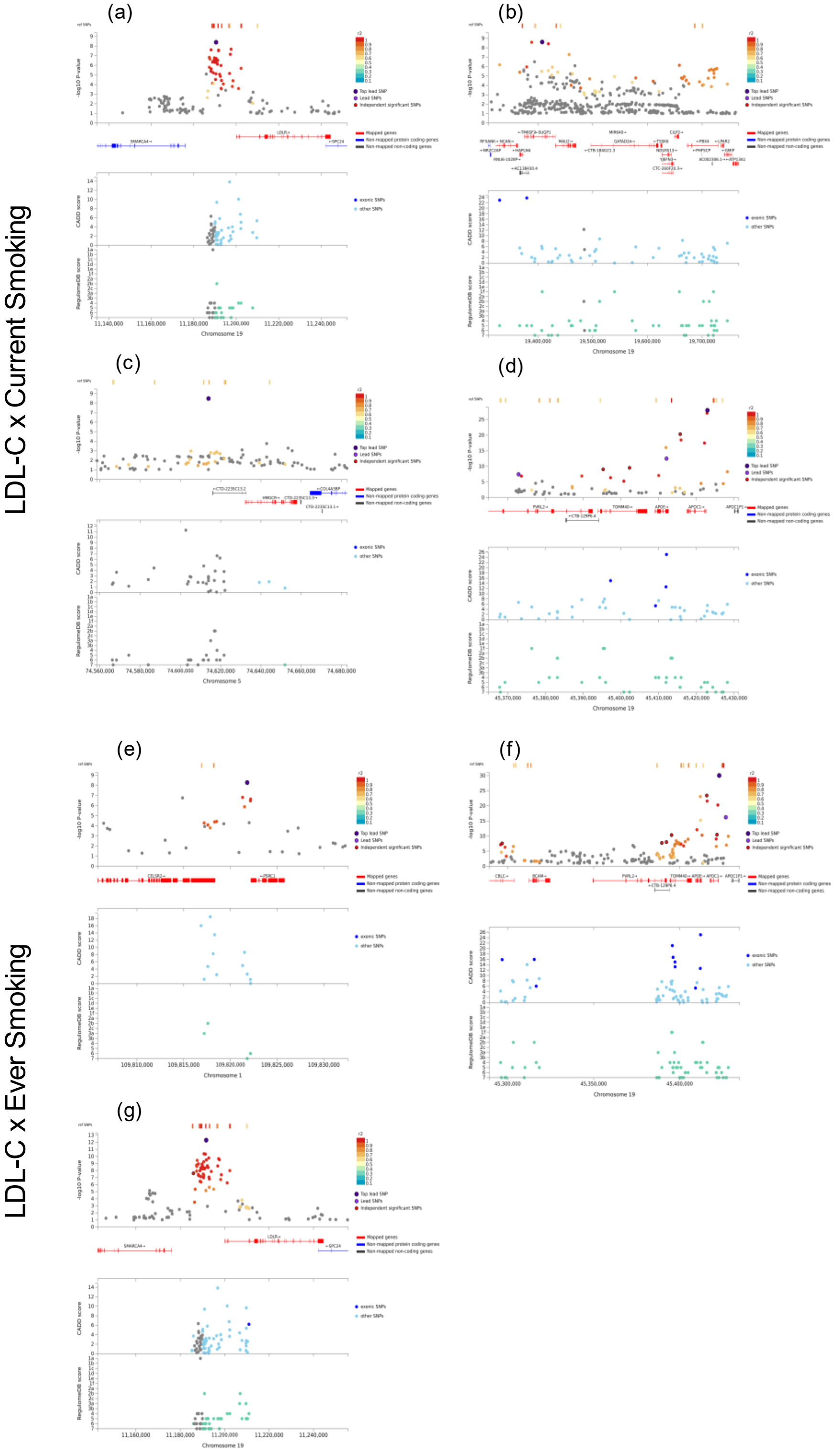

Supplementary Figure 11.3. Zoomed locus-specific plots for the GxE loci identified by T_{MR-GxE} for HDL-C. In each panel, top is -log10(P-value) and bottom is the corresponding CADD and RegulomDB score generated by software FUMA. (a) *LIPC/ALDH1A2*, Current Drinking. (b) *DDX28/DUS2/NFATC3*, Current Drinking (c) *CETP*, Current Drinking. (d) *GALNT2*, Current drinking. (e) *RPL5P26*, regular drinking. (f) *LIPC/ALDH1A2*, Regular drinking. (g) *CETP*, Regular Drinking. (h) *LPL*, Regular Drinking.

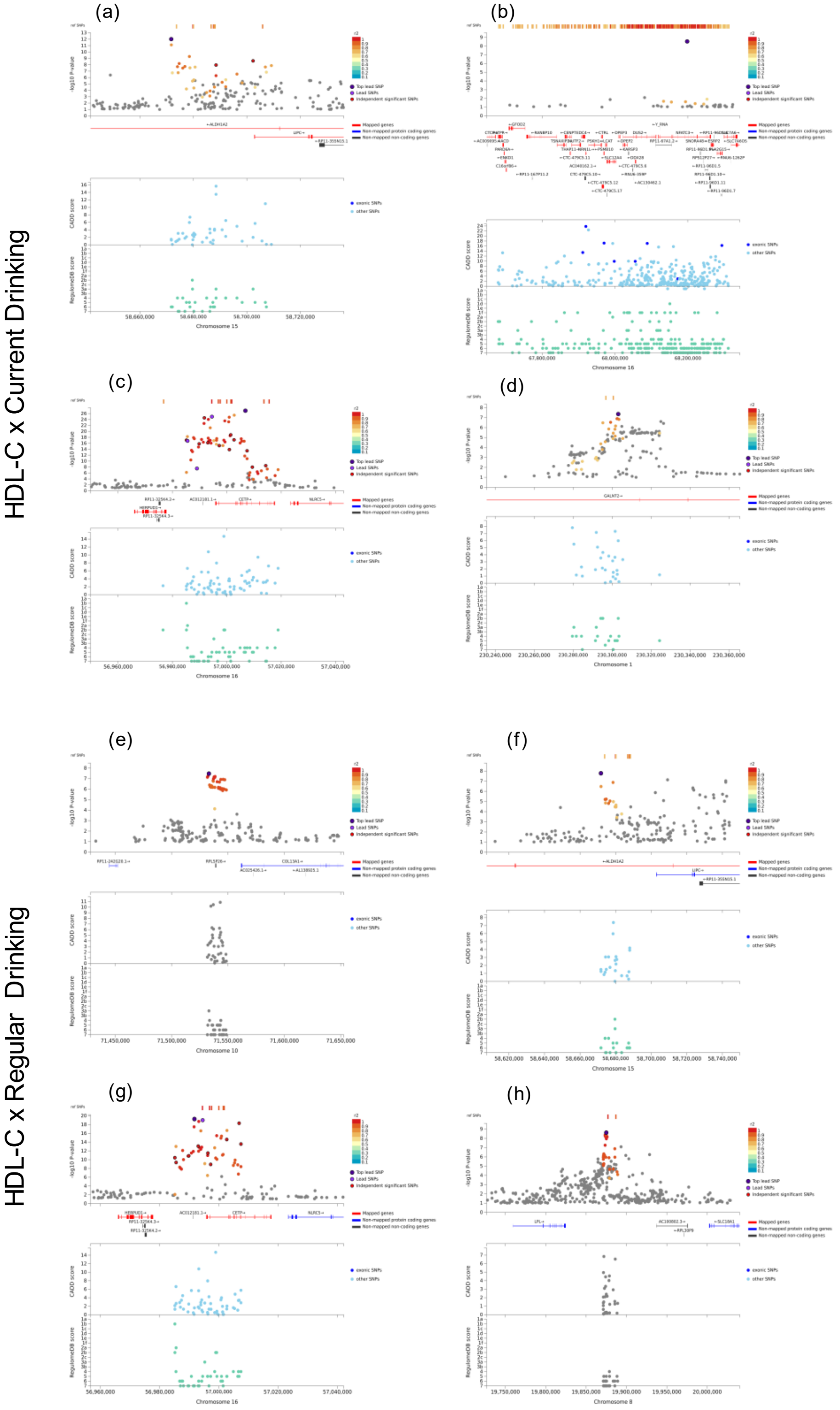

Supplementary Figure 11.4. Zoomed locus-specific plots for the GxE loci identified by T_{MR-GxE} for HDL-C. In each panel, top is -log10(P-value) and bottom is the corresponding CADD and RegulomDB score generated by software FUMA. (a) *CETP*, Current Smoking. (b) *LIPC/ALDH1A2*, Current Smoking. (c) *LPL*, Current smoking. (d) *APOC1*, current smoking.

HDL-C x Current Smoking

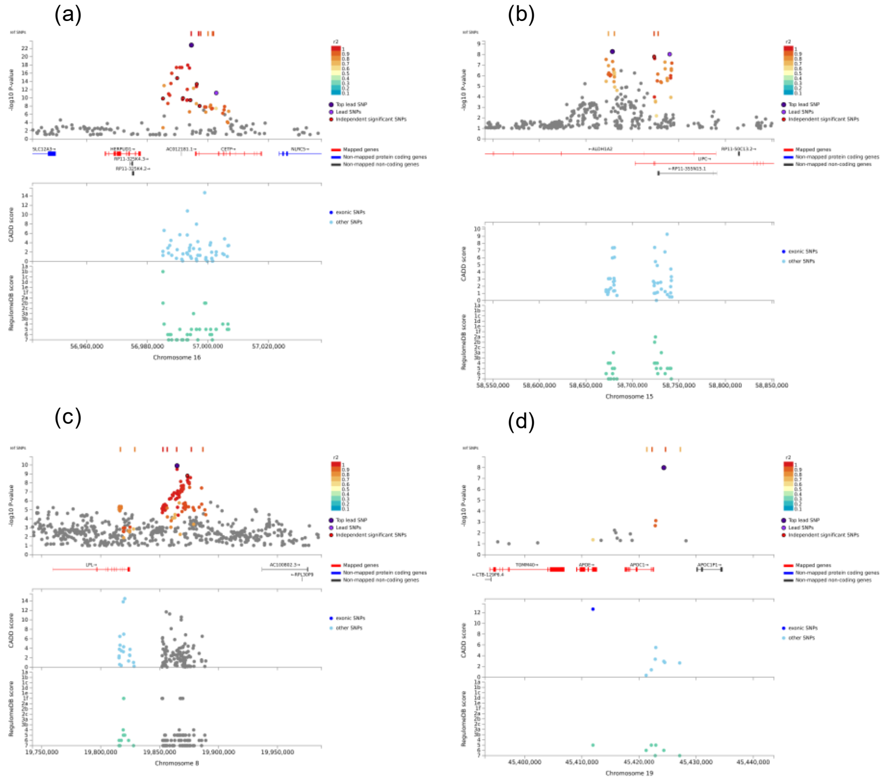

Supplementary Figure 11.5. Zoomed locus-specific plots for the GxE loci identified by T_{MR-GxE} for HDL-C. In each panel, top is -log10(P-value) and bottom is the corresponding CADD and RegulomDB score generated by software FUMA. (a) *LIPC/ALDH1A2*, Ever Smoking. (b) *DDX28/DUS2/NFATC3*, Ever smoking. (c) *LPL*, Ever smoking. (d) *CETP*, ever smoking.

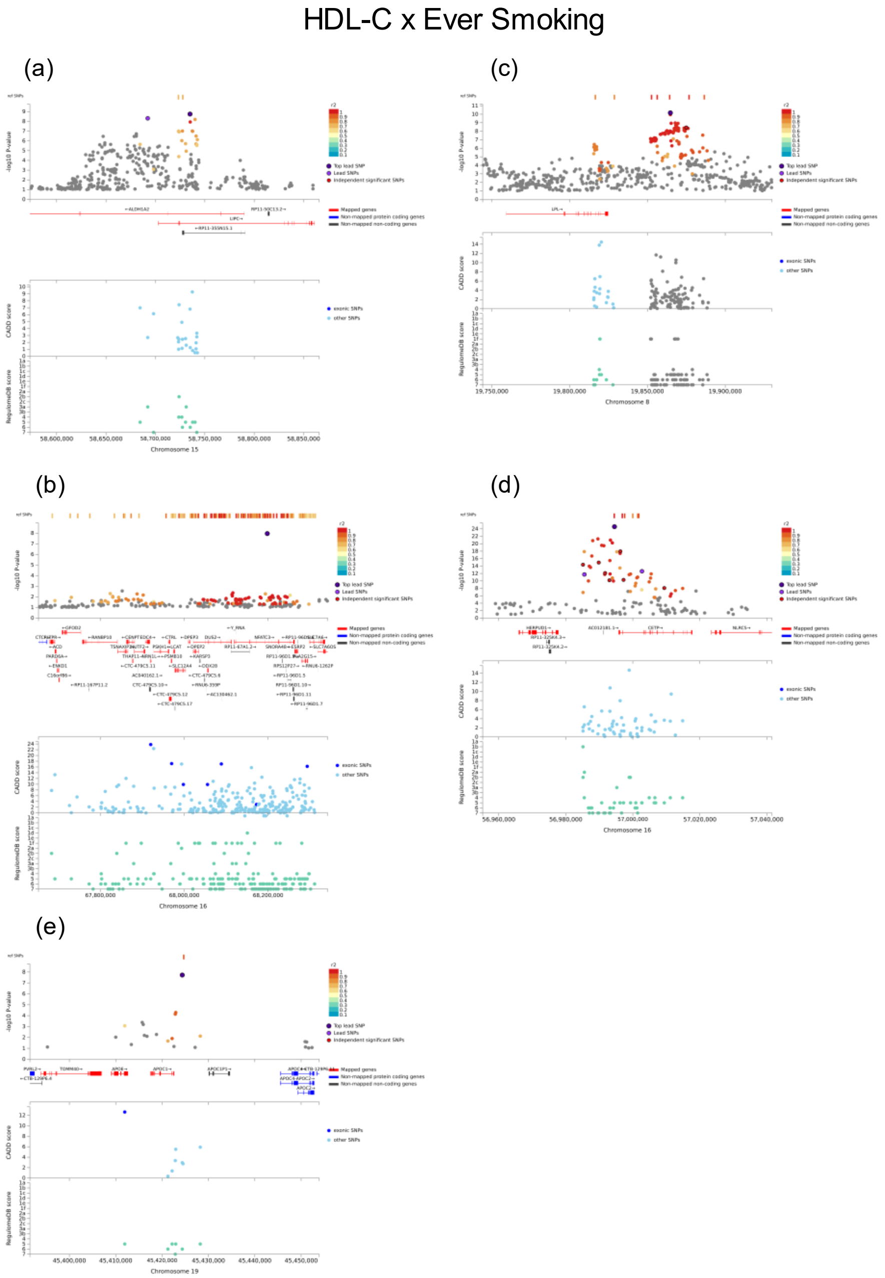

Supplementary Figure 11.6. Zoomed locus-specific plots for the GxE loci identified by T_{MR-GxE} for TG. In each panel, top is -log10(P-value) and bottom is the corresponding CADD and RegulomDB score generated by software FUMA. (a) *LPL*, Current Drinking. (b) *BUD13*, Current Drinking (c) *APOE/APOC1*, Current Drinking. (d) *AC091114.1*, Regular drinking. (e) *BUD13*, regular drinking. (f) *APOE/APOC1*, Regular drinking. (g) *LPL*, Regular Drinking.

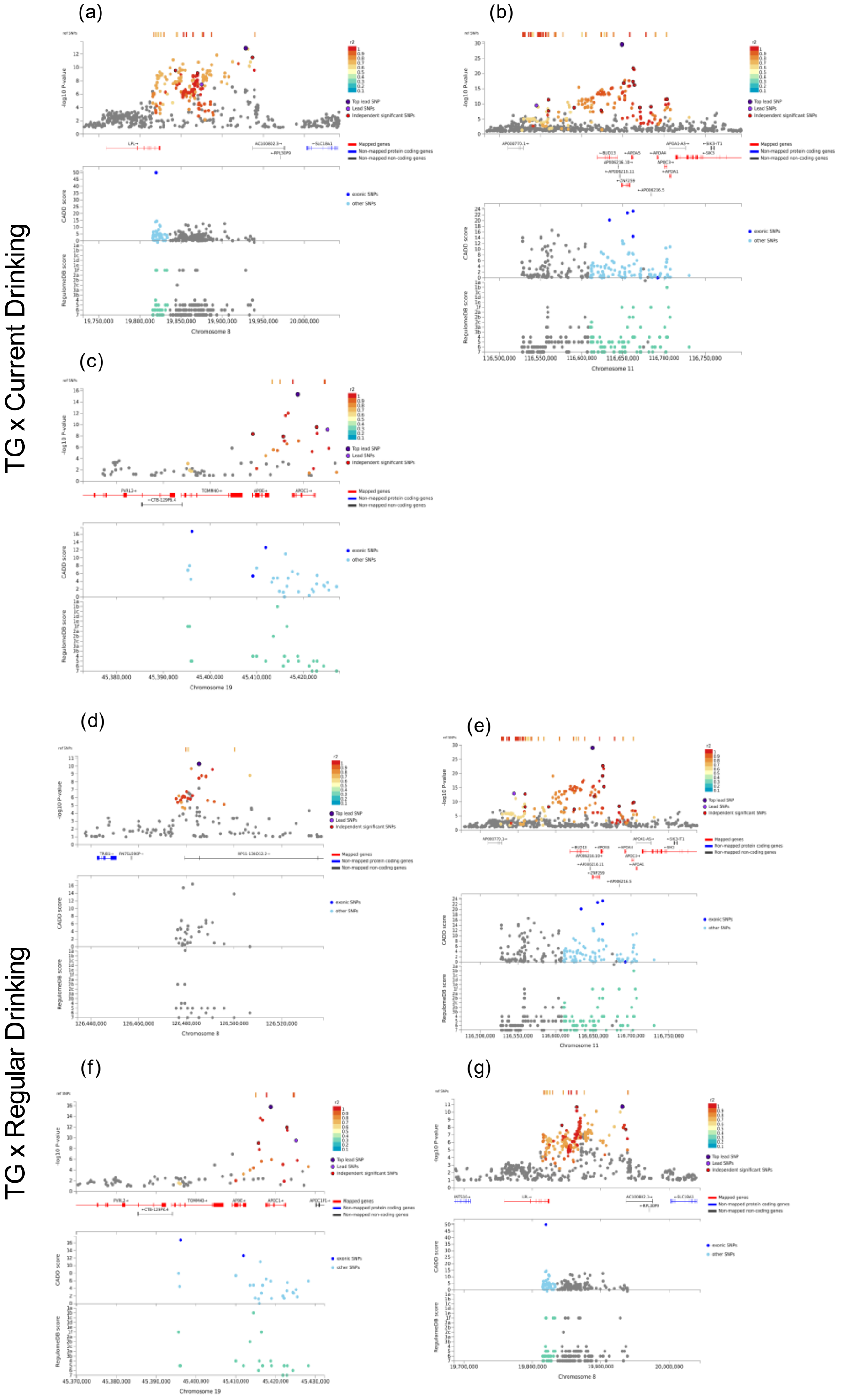

Supplementary Figure 11.7. Zoomed locus-specific plots for the GxE loci identified by T_{MR-GxE} for TG. In each panel, top is -log10(P-value) and bottom is the corresponding CADD and RegulomDB score generated by software FUMA. (a) *ZNF512*, Current Smoking. (b) *AC091114.1*, Current Smoking (c) *BUD13*, Current Smoking (d) *LPL*, Current Smoking. (e) *APOE/APOC1*, Current Smoking.

TG x Current Smoking

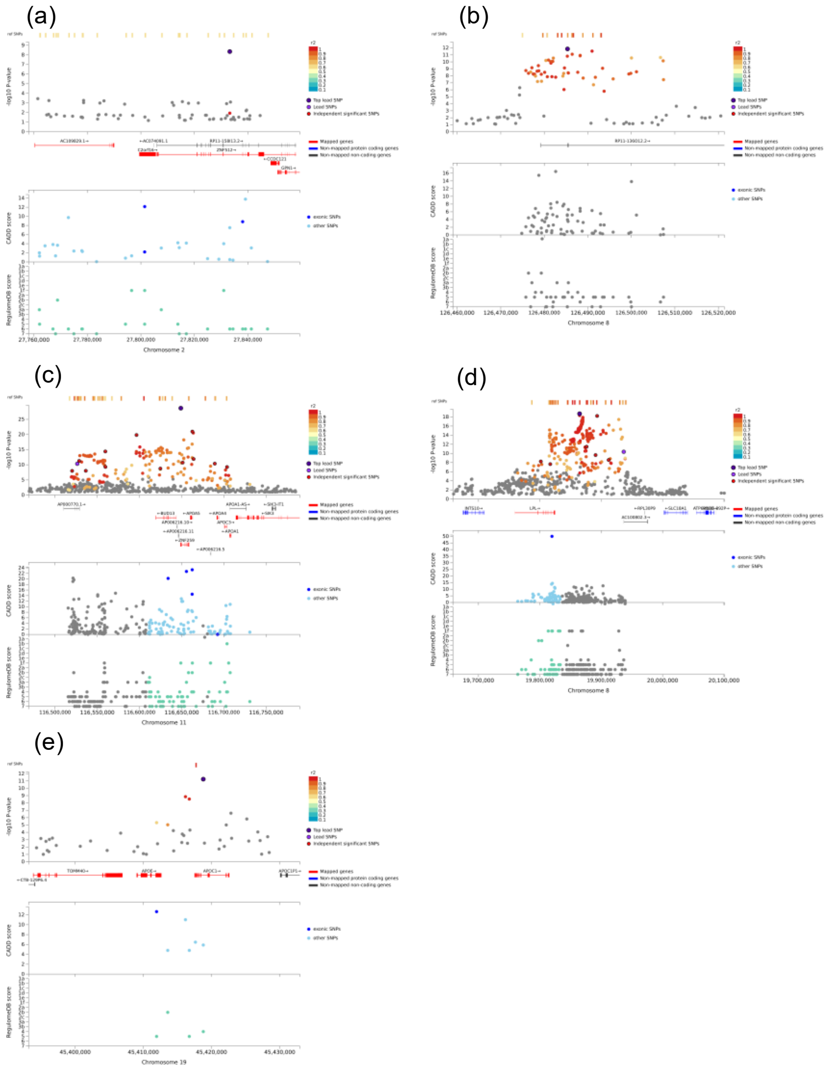

Supplementary Figure 11.8. Zoomed locus-specific plots for the GxE loci identified by T_{MR-GxE} for TG. In each panel, top is -log10(P-value) and bottom is the corresponding CADD and RegulomDB score generated by software FUMA. (a) *BUD13*, Ever Smoking. (b) *APOE/APOC1*, Ever Smoking. (c) *AC091114.1*, Ever Smoking. (d) *LPL*, Ever Smoking.

TG x Ever Smoking

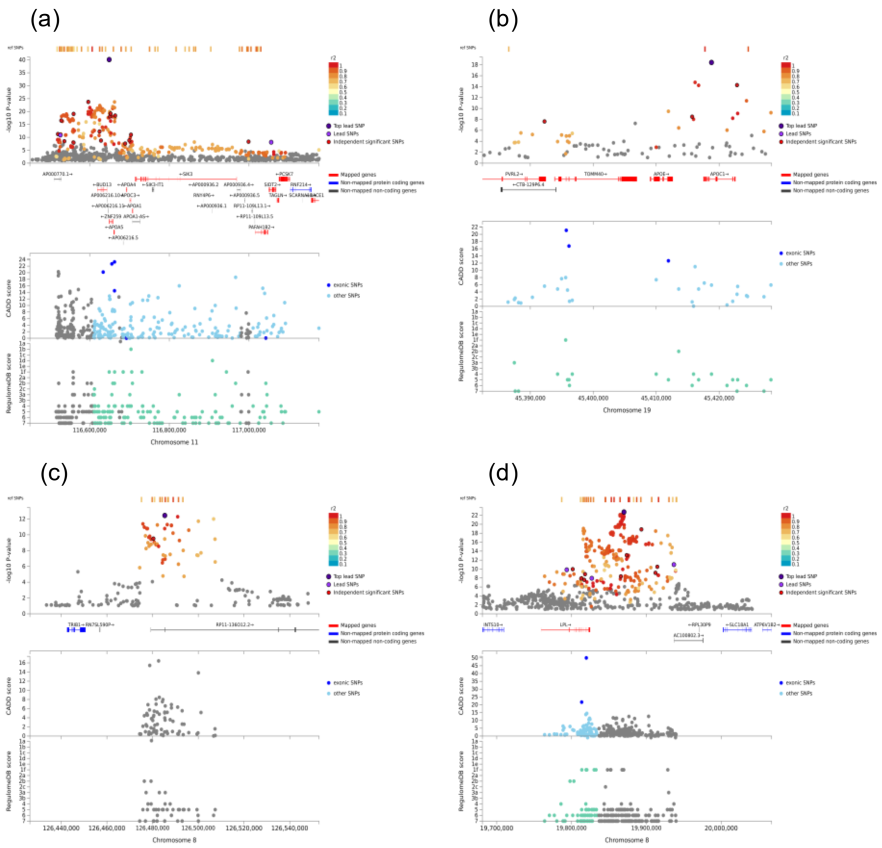

Supplementary Figure 12. The circle Manhattan plots of $G \times E$ by $T_{MR\ GxE}$ for LDL-C, HDL-C and TG in ancestry specific analysis.

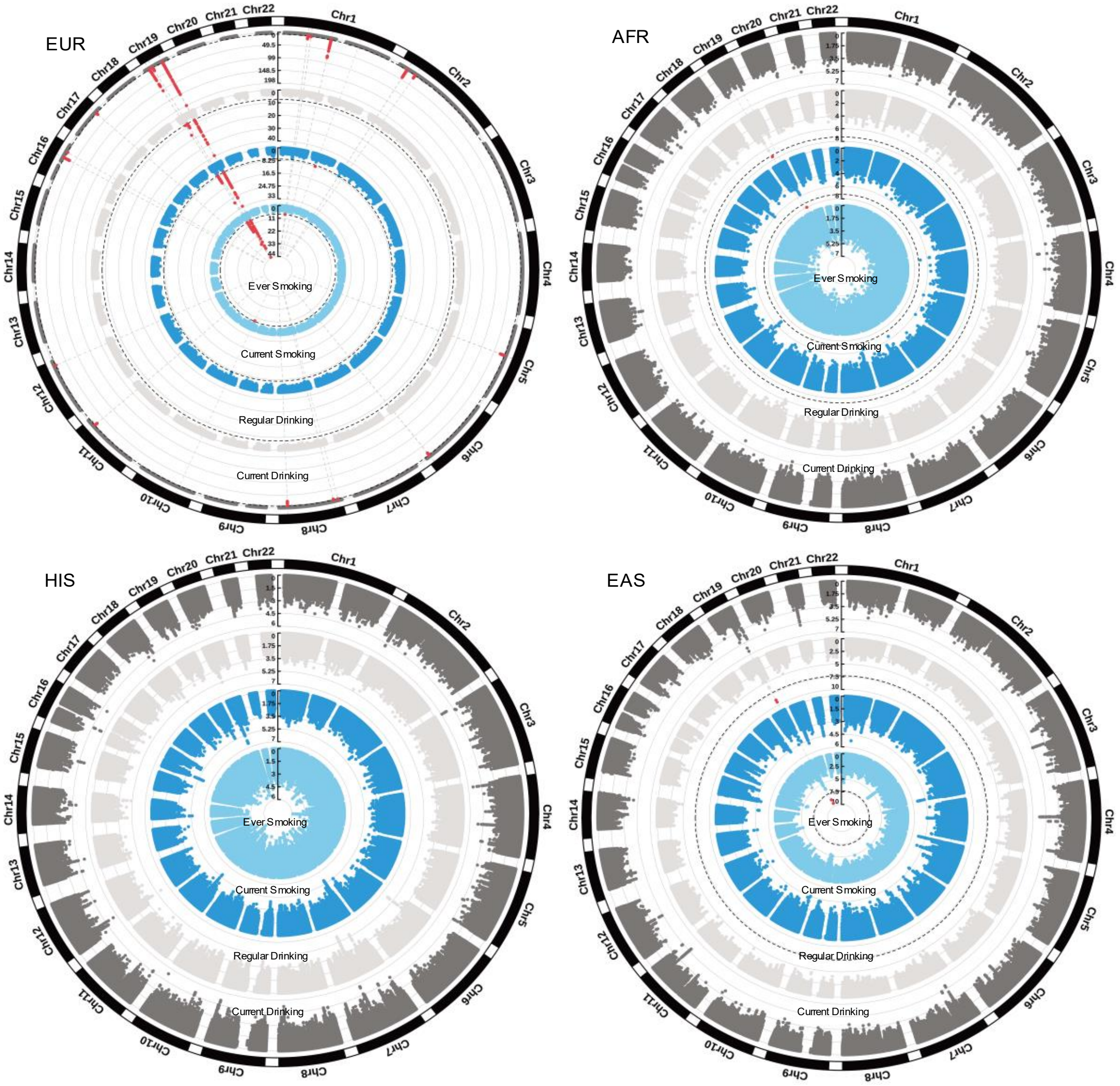

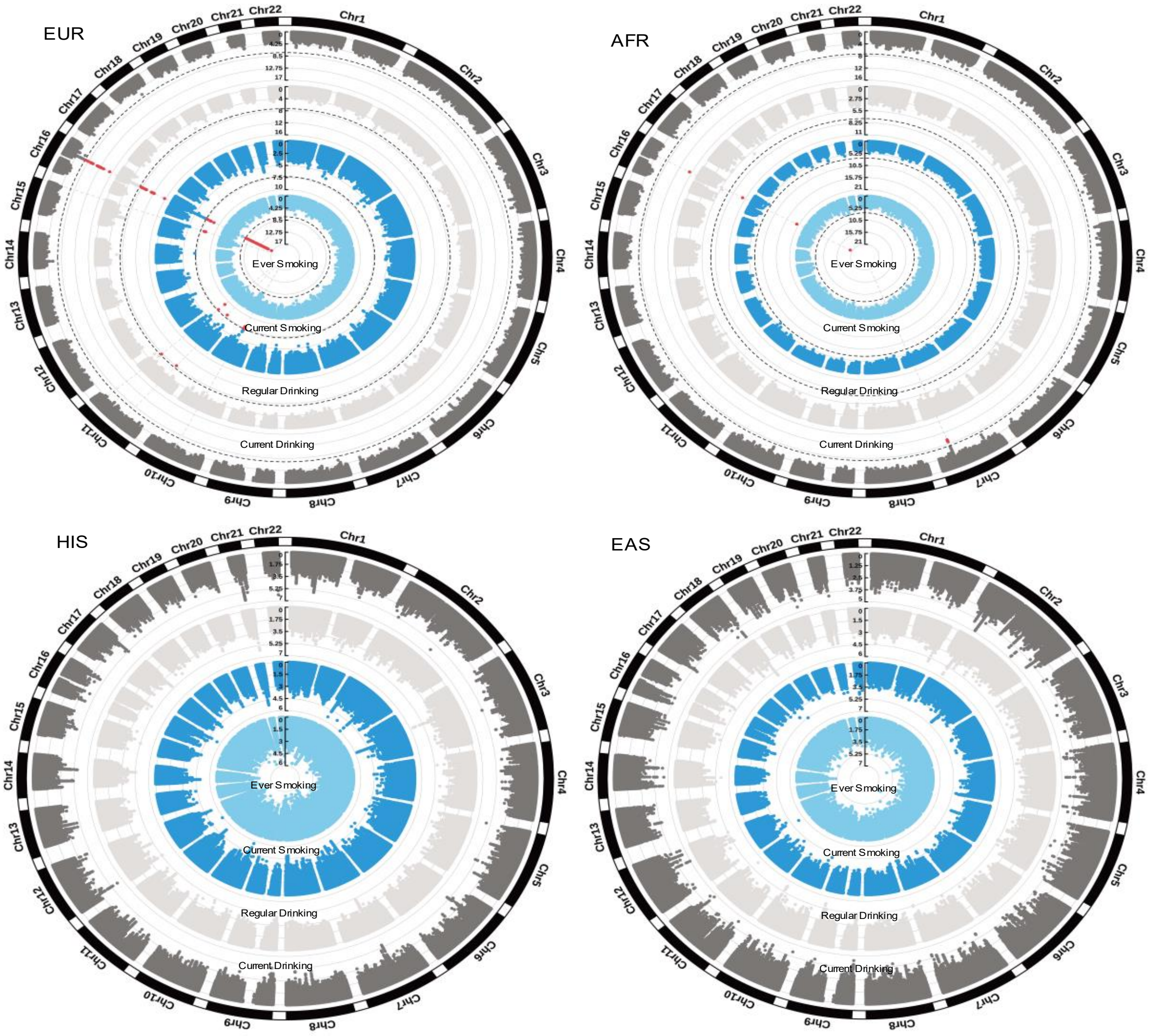
EUR
Chr1
Chr2
Chr3
Chr4
Chr5
Chr6
Chr7
Chr8
Chr9
Chr10
Chr11
Chr12
Chr13
Chr14
Chr15
Chr16
Chr17
Chr18
Chr19
Chr20
Chr21
Chr22
Ever Smoking
Current Smoking
Regular Drinking
Current Drinking
AFR
Chr1
Chr2
Chr3
Chr4
Chr5
Chr6
Chr7
Chr8
Chr9
Chr10
Chr11
Chr12
Chr13
Chr14
Chr15
Chr16
Chr17
Chr18
Chr19
Chr20
Chr21
Chr22
Ever Smoking
Current Smoking
Regular Drinking
Current Drinking
HIS
Chr1
Chr2
Chr3
Chr4
Chr5
Chr6
Chr7
Chr8
Chr9
Chr10
Chr11
Chr12
Chr13
Chr14
Chr15
Chr16
Chr17
Chr18
Chr19
Chr20
Chr21
Chr22
Ever Smoking
Current Smoking
Regular Drinking
Current Drinking
EAS
Chr1
Chr2
Chr3
Chr4
Chr5
Chr6
Chr7
Chr8
Chr9
Chr10
Chr11
Chr12
Chr13
Chr14
Chr15
Chr16
Chr17
Chr18
Chr19
Chr20
Chr21
Chr22
Ever Smoking
Current Smoking
Regular Drinking
Current Drinking

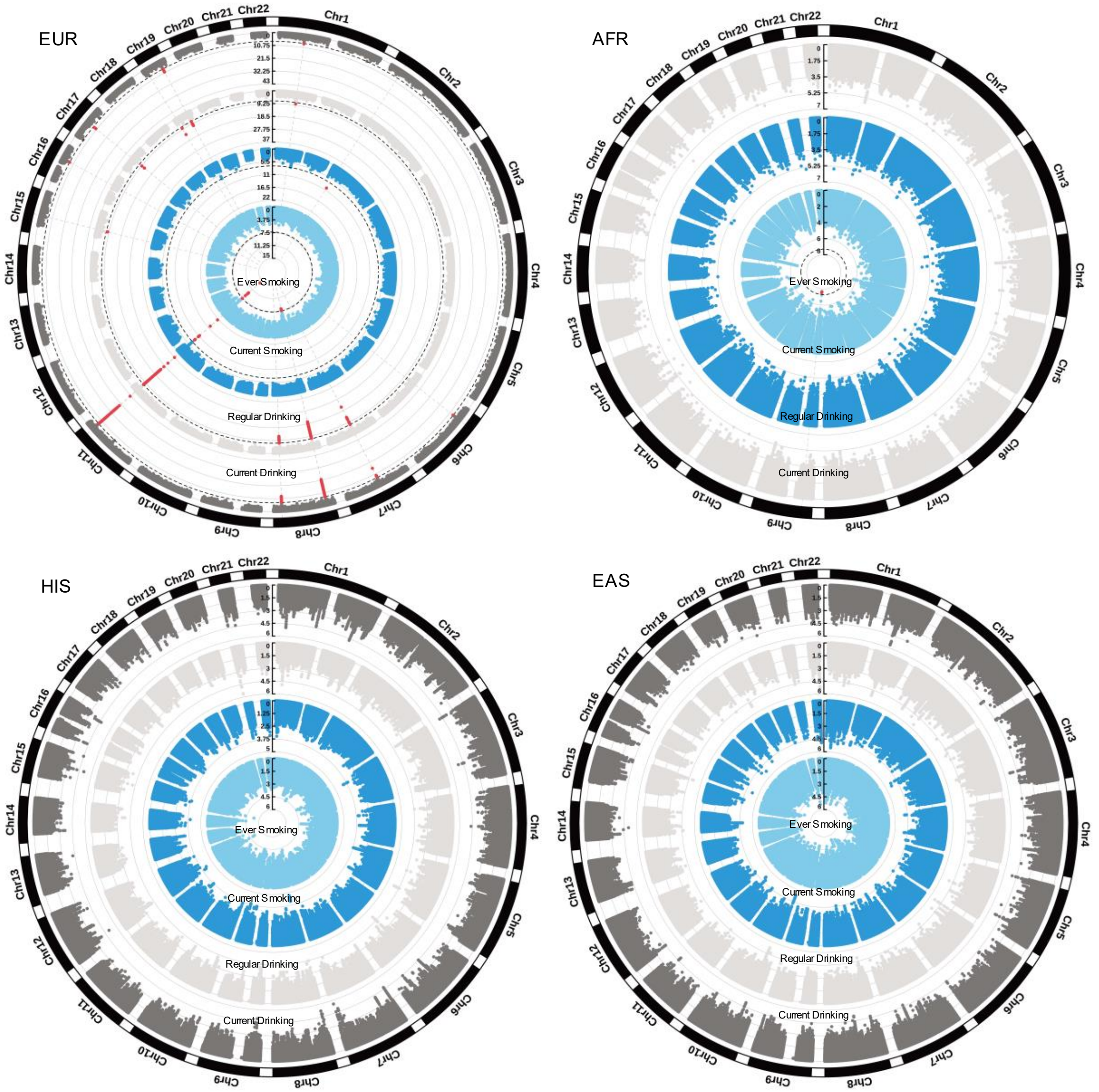
EUR
AFR
HIS
EAS
Chr1
Chr2
Chr3
Chr4
Chr5
Chr6
Chr7
Chr8
Chr9
Chr10
Chr11
Chr12
Chr13
Chr14
Chr15
Chr16
Chr17
Chr18
Chr19
Chr20
Chr21
Chr22
Ever Smoking
Current Smoking
Regular Drinking
Current Drinking

Supplementary Figure 13.1. (a)-(b): the MAGMA tissue enrichment analysis across 30 general tissue types and 54 specific tissue types from GTEx, respectively, for LDL-C and Current Drinking based on T_{MR-GxE} test; (c)-(d): Differentially expressed genes across 30 general tissue types and 54 specific tissue types, respectively, for LDL-C and Current Drinking based on T_{MR-GxE} test. (e)-(h): the counterparts of (a)-(d) for LDL-C and Regular Drinking.

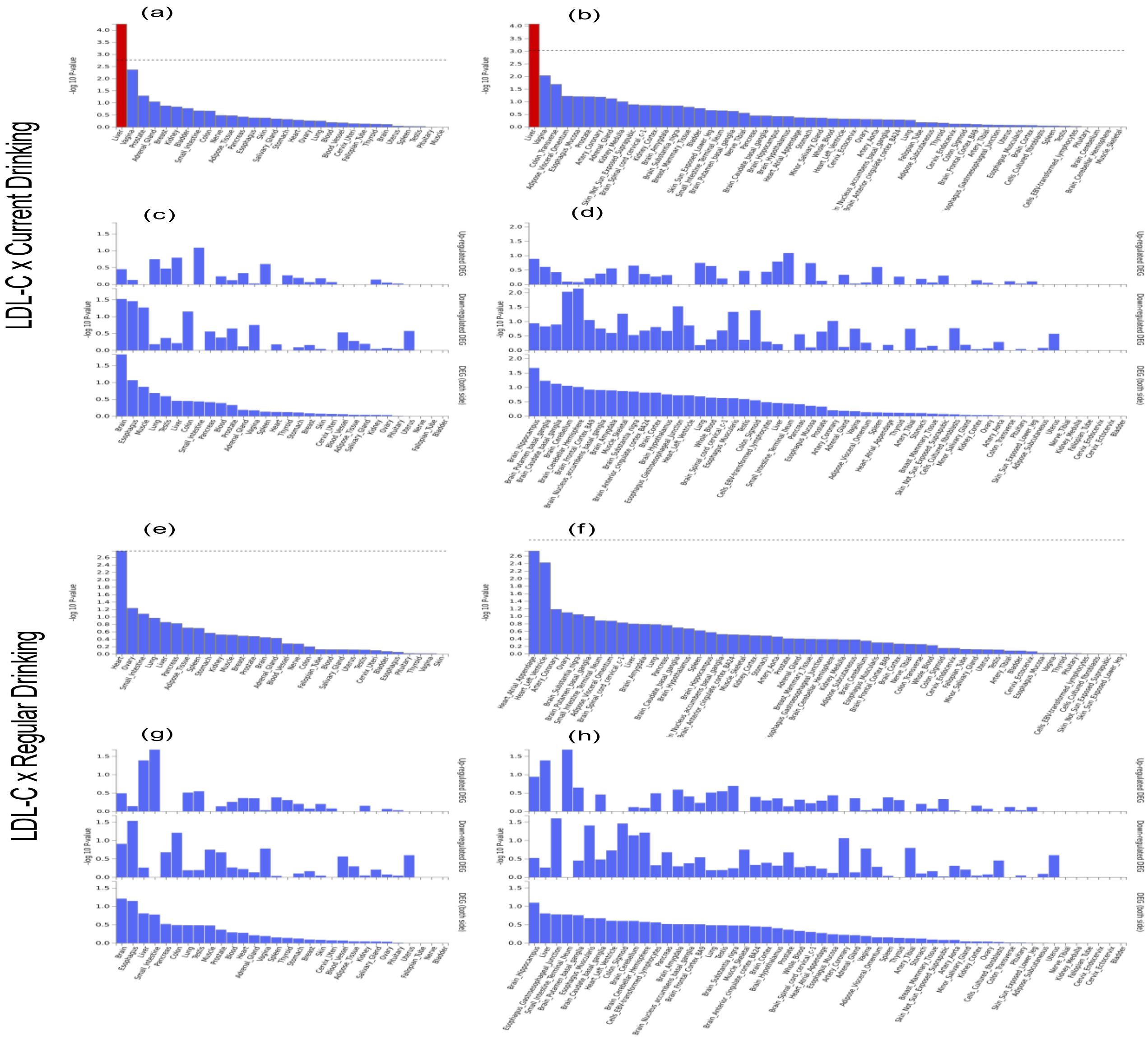

Supplementary Figure 13.2. (a)-(b): the MAGMA tissue enrichment analysis across 30 general tissue types and 54 specific tissue types from GTEx, respectively, for LDL-C and Current smoking based on T_{MR-GxE} test; (c)-(d): Differentially expressed genes across 30 general tissue types and 54 specific tissue types, respectively, for LDL-C and Current smoking based on T_{MR-GxE} test. (e)-(h): the counterparts of (a)-(d) for LDL-C and Ever Smoking.

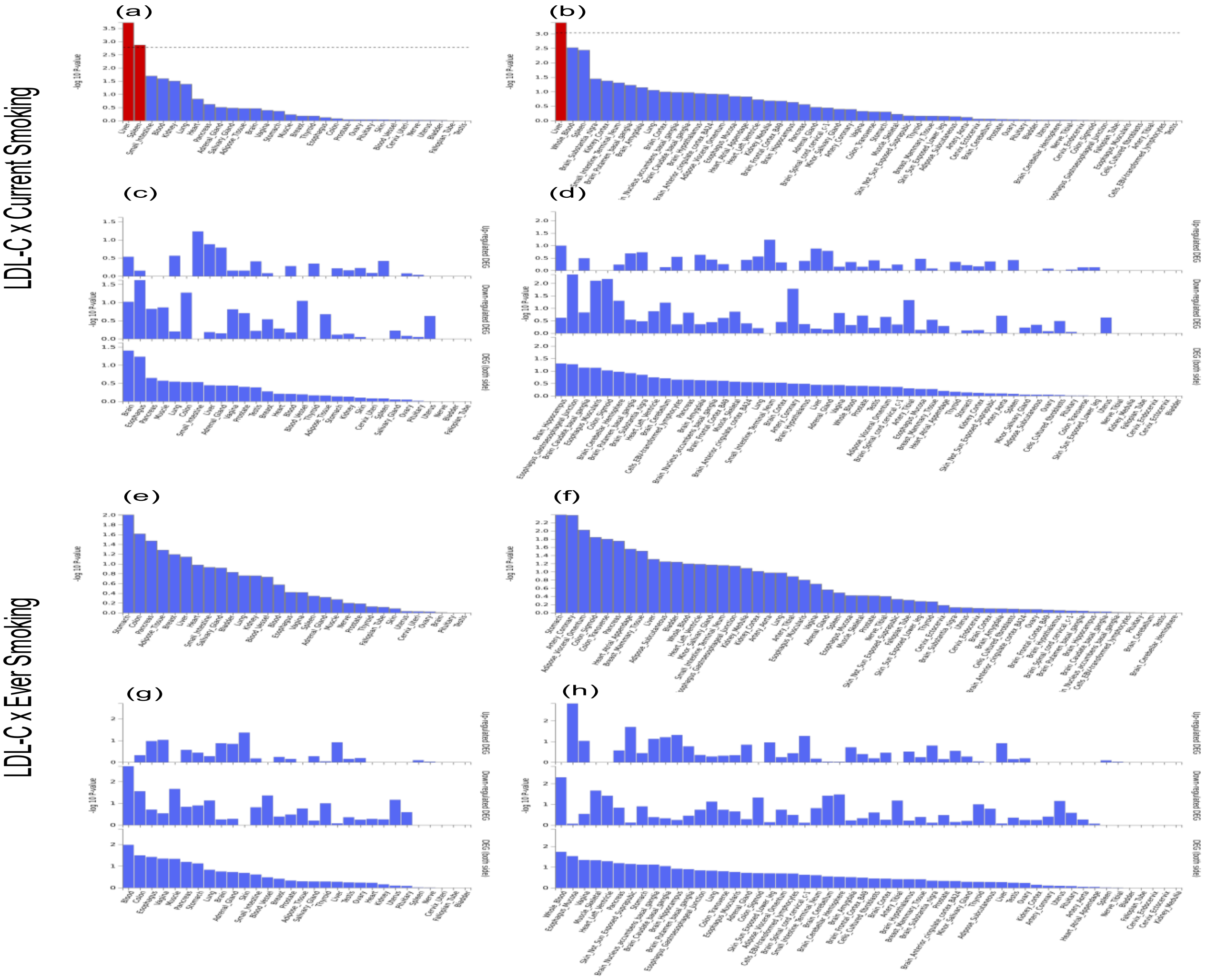

Supplementary Figure 13.3. (a)-(b): the MAGMA tissue enrichment analysis across 30 general tissue types and 54 specific tissue types from GTEx, respectively, for HDL-C and Current Drinking based on T_{MR-GxE} test; (c)-(d): Differentially expressed genes across 30 general tissue types and 54 specific tissue types, respectively, for HDL-C and Current Drinking based on T_{MR-GxE} test. (e)-(h): the counterparts of (a)-(d) for HDL-C and Regular Drinking.

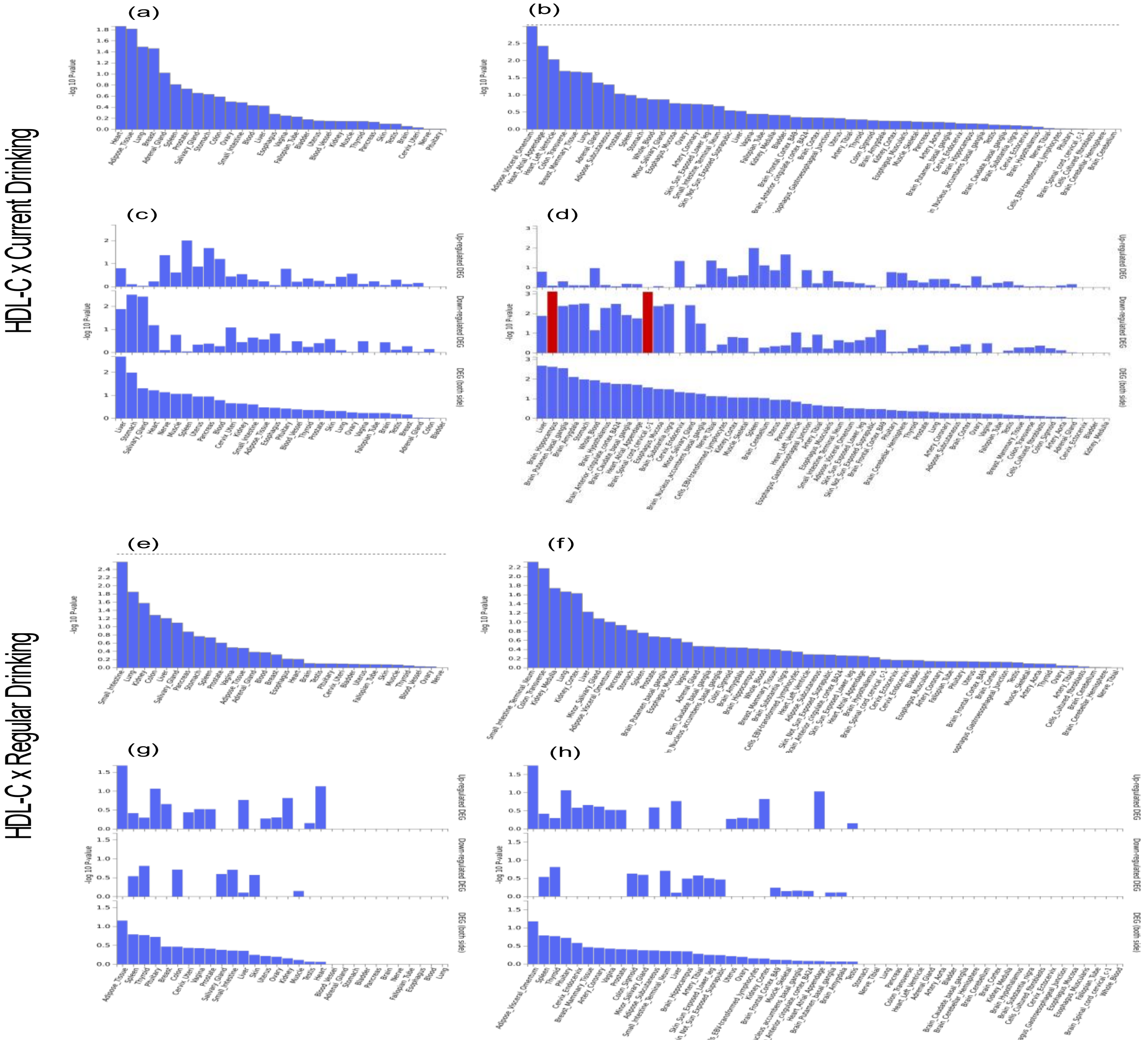

Supplementary Figure 13.4. (a)-(b): the MAGMA tissue enrichment analysis across 30 general tissue types and 54 specific tissue types from GTEx, respectively, for HDL-C and Current smoking based on T_{MR-GxE} test; (c)-(d): Differentially expressed genes across 30 general tissue types and 54 specific tissue types, respectively, for HDL-C and Current smoking based on T_{MR-GxE} test. (e)-(h): the counterparts of (a)-(d) for HDL-C and Ever Smoking, respectively.

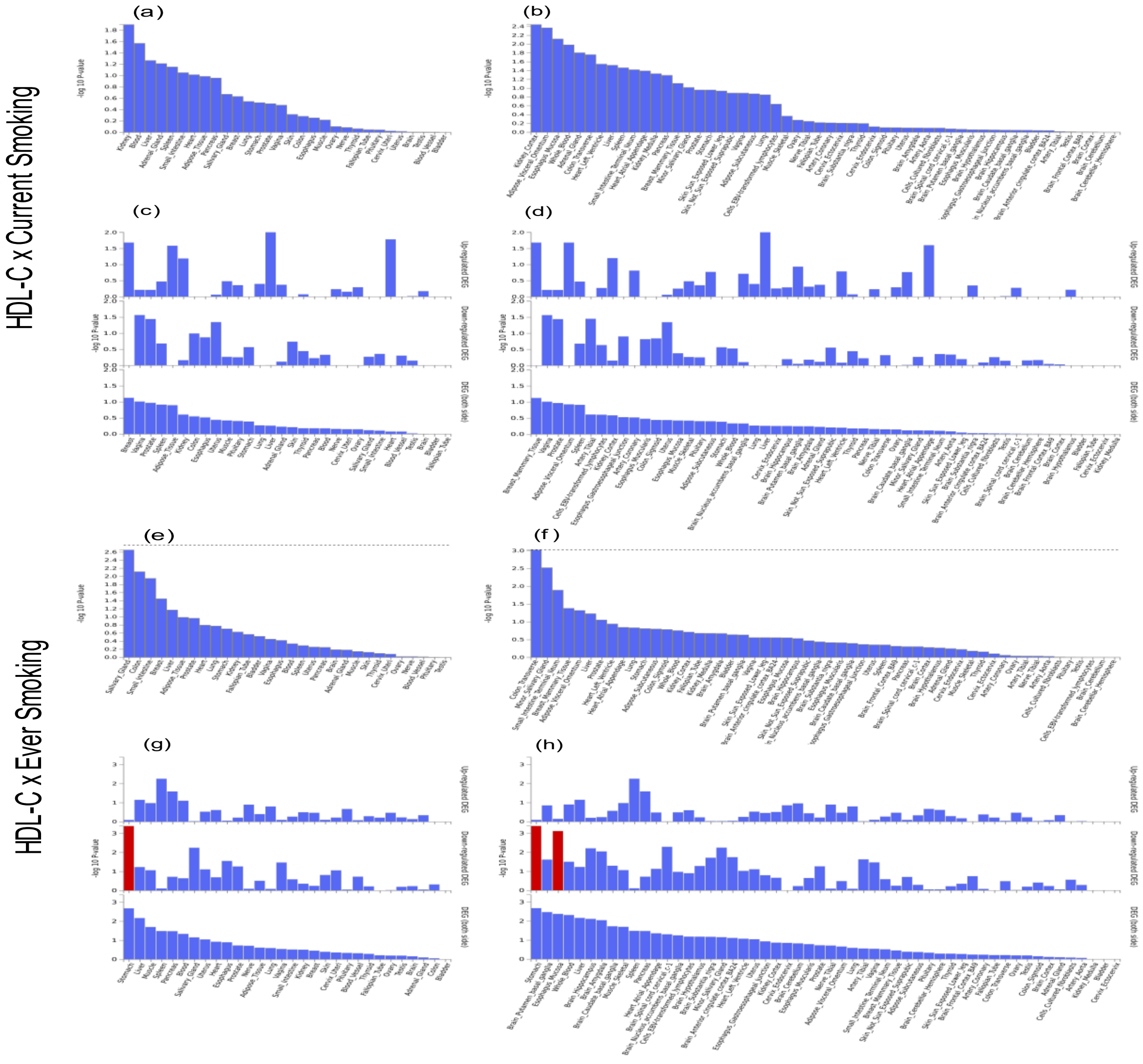

Supplementary Figure 13.5. (a)-(b): the MAGMA tissue enrichment analysis across 30 general tissue types and 54 specific tissue types from GTEx, respectively, for TG and Current Drinking based on T_{MR-GxE} test; (c)-(d): Differentially expressed genes across 30 general tissue types and 54 specific tissue types, respectively, for TG and Current Drinking based on T_{MR-GxE} test. (e)-(h): the counterparts of (a)-(d) for TG and Regular Drinking.

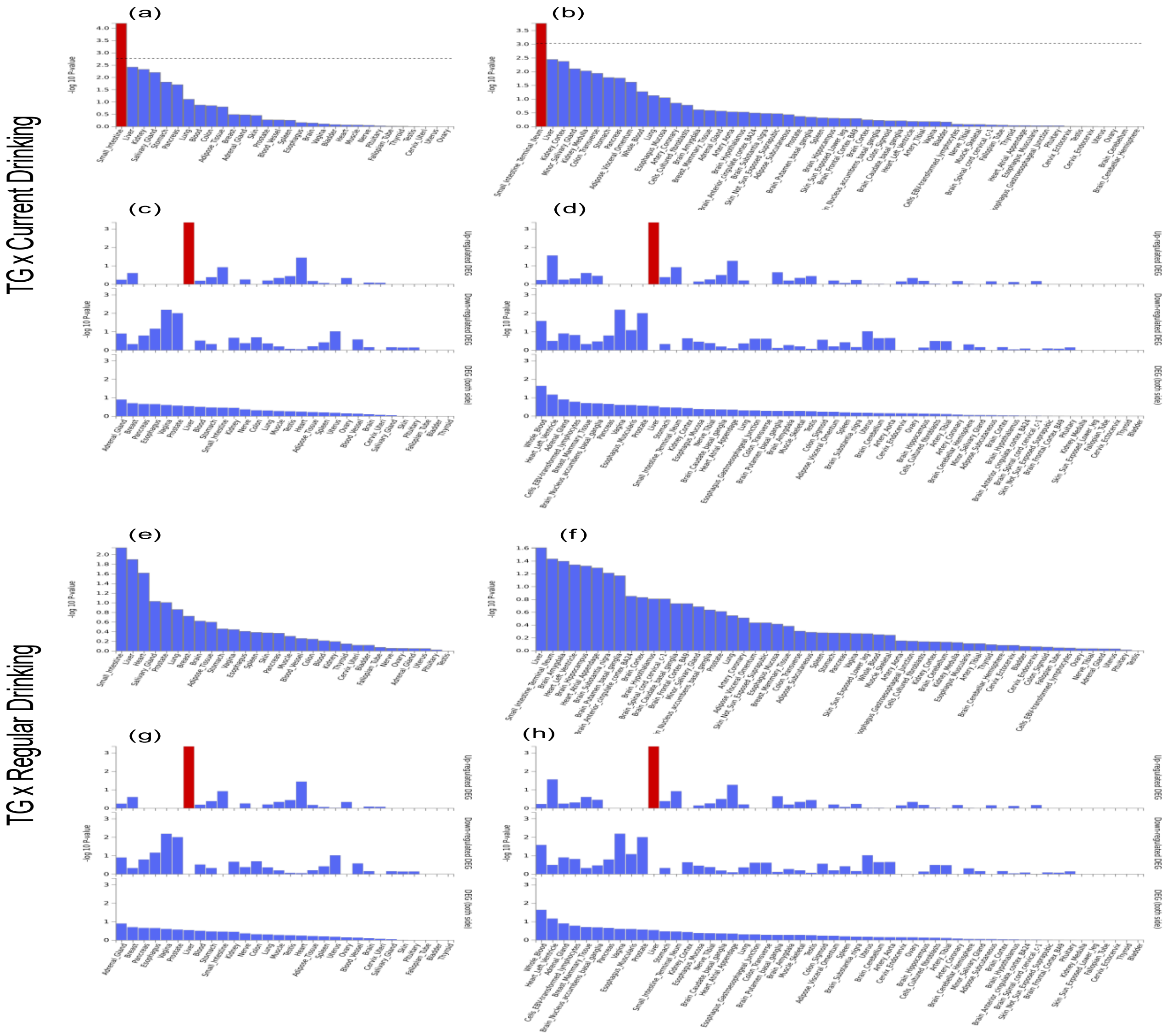

Supplementary Figure 13.6. (a)-(b): the MAGMA tissue enrichment analysis across 30 general tissue types and 54 specific tissue types from GTEx, respectively, for TG and Current Smoking based on T_{MR-GxE} test; (c)-(d): Differentially expressed genes across 30 general tissue types and 54 specific tissue types, respectively, for TG and Current Smoking based on T_{MR-GxE} test. (e)-(h): the counterparts of (a)-(d) for TG and Ever Smoking.

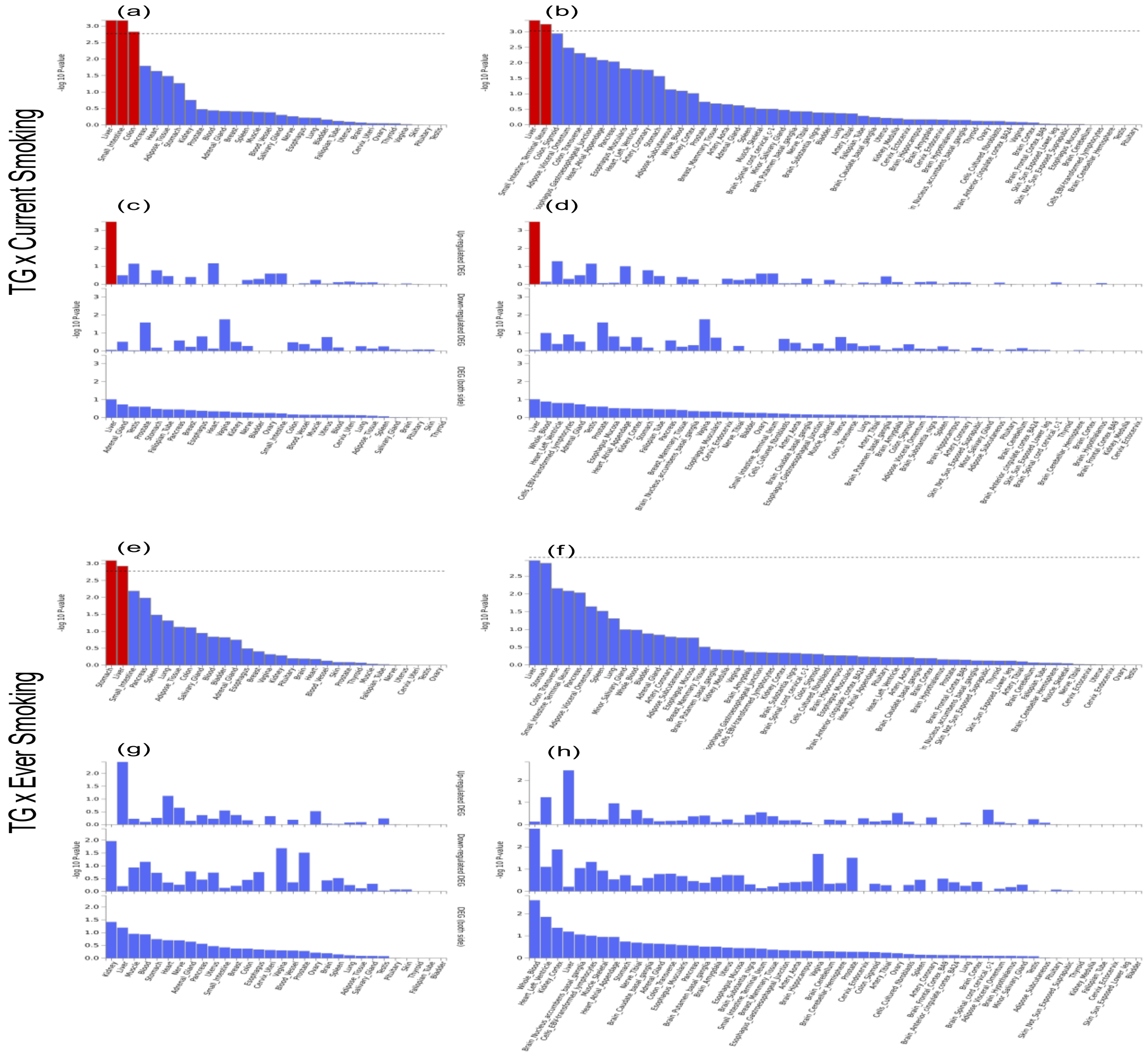

Supplementary Figure 14. Colocalization between TM6SF2 gene and LDL-C x Current Smoking in Lung tissue. rs1009136 is a potential causal SNP with CLPP 0.5216.

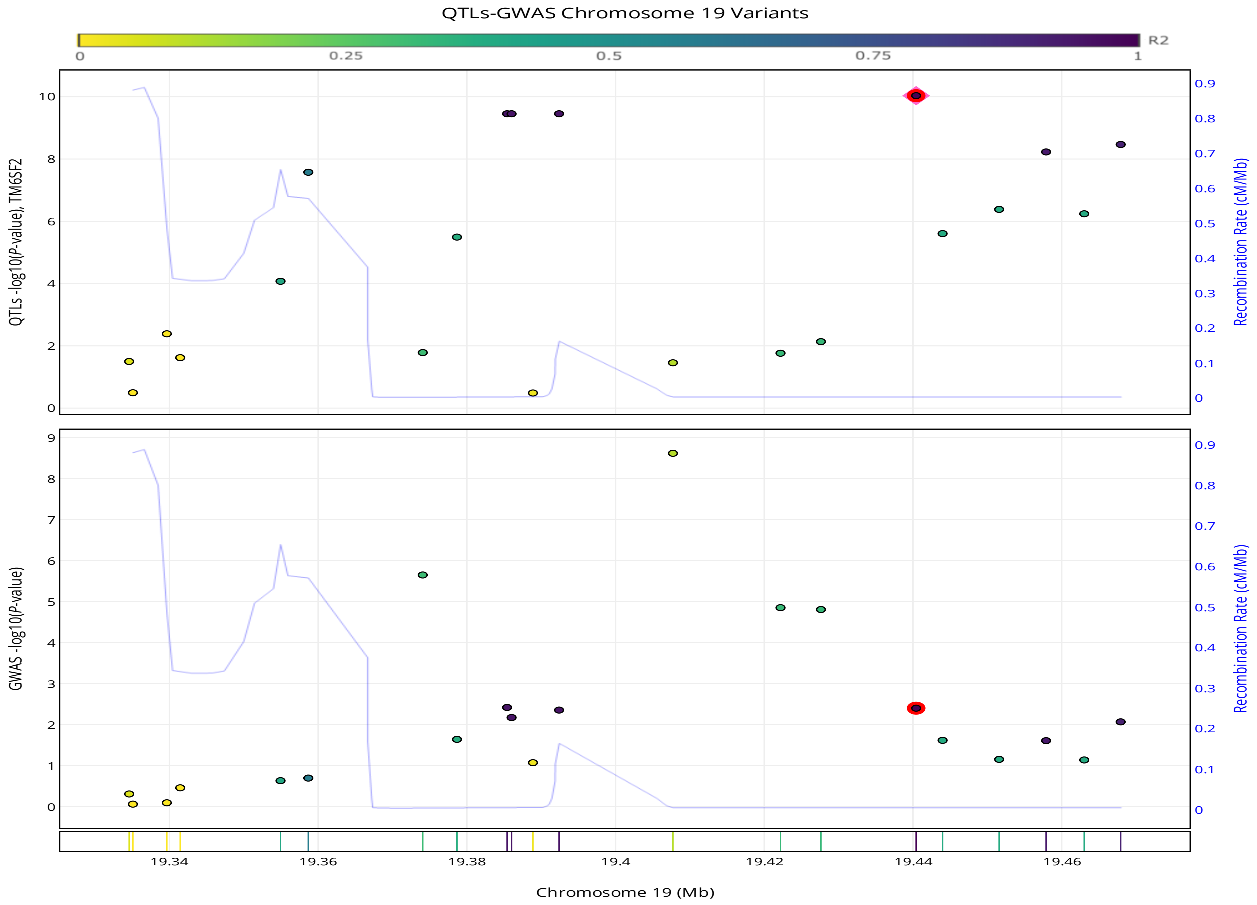

Colocalization between MAU2 gene and LDL-C x Current Drinking in Liver tissue. rs10401969 is a potential causal SNP with CLPP 0.8211.

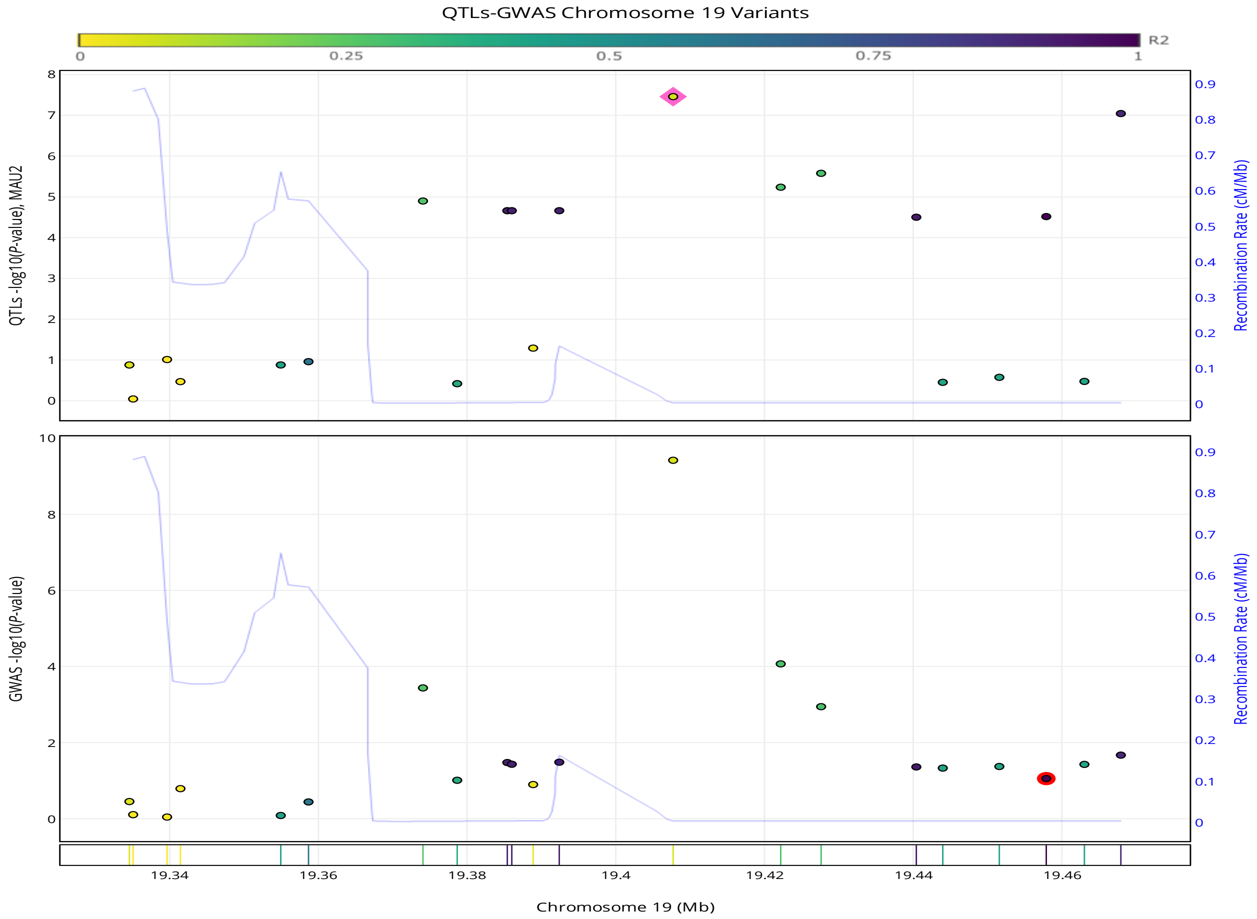

Colocalization between OPA3 gene and LDL-C x Current Drinking in Liver tissue. rs4420638 is a potential causal SNP with CLPP 0.3284.

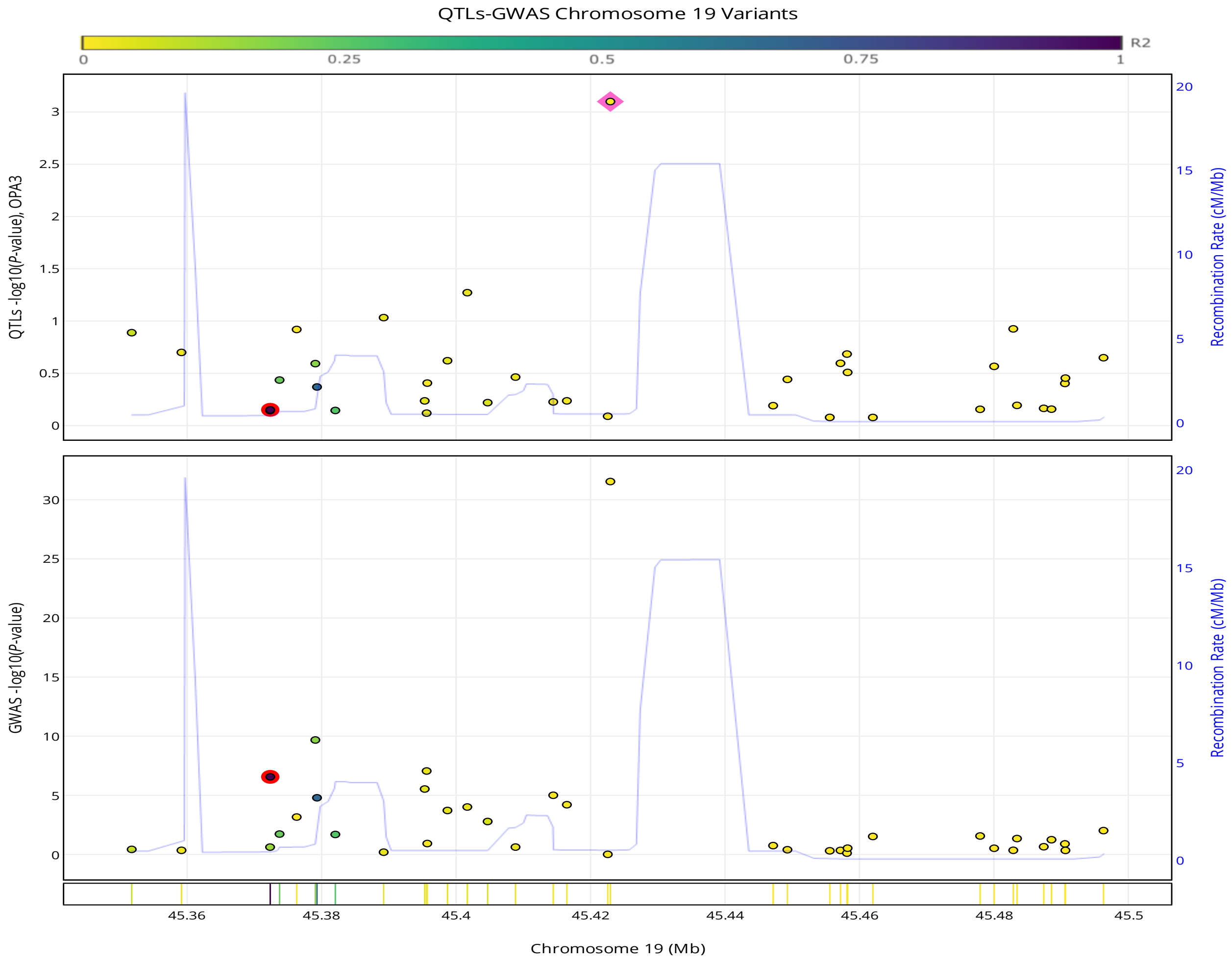

Colocalization between APOC1P1 gene and TG x Regular Drinking in Liver tissue. rs584007 is a potential causal SNP with CLPP 0.1479.

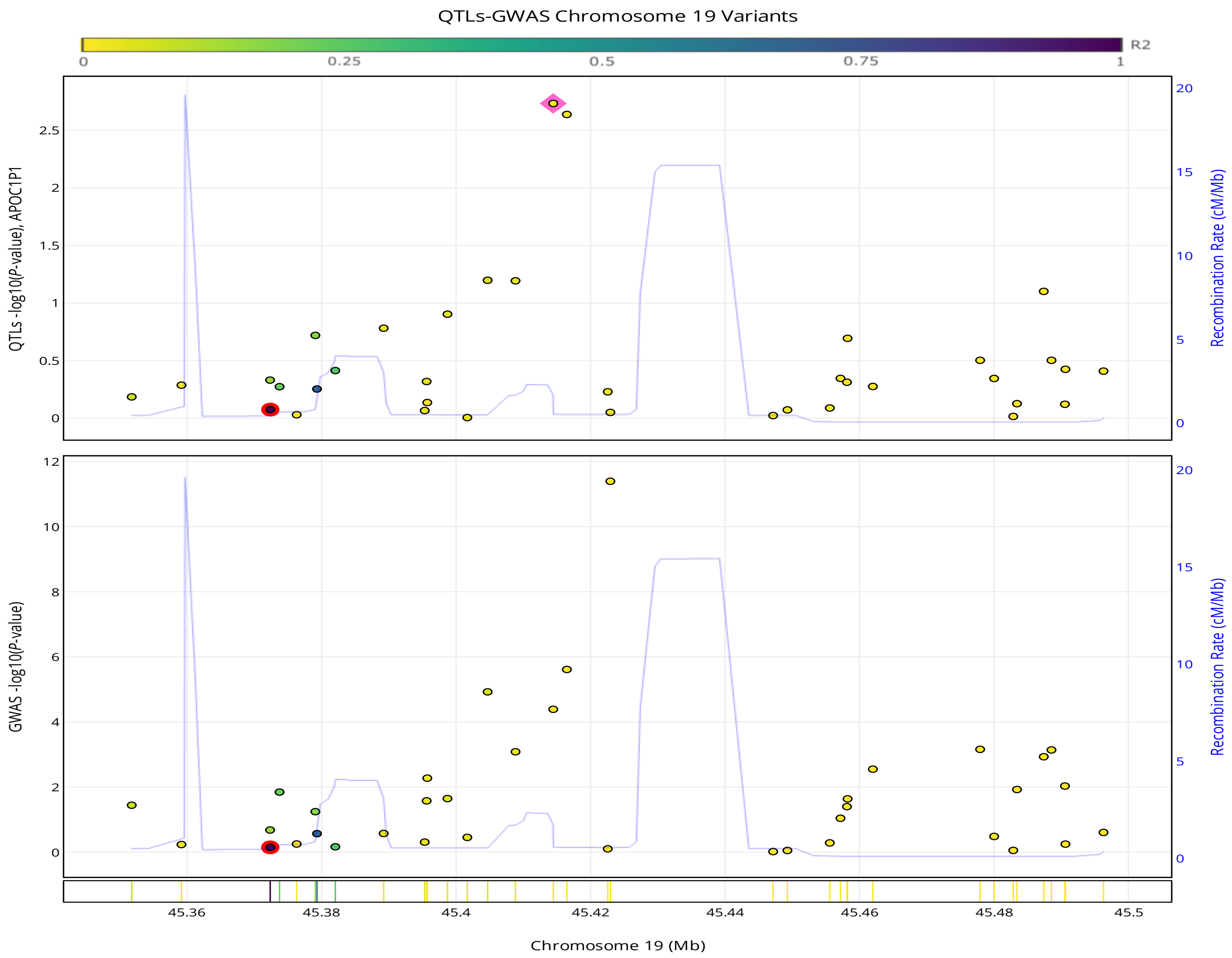

Colocalization between APOC1P1 gene and TG x Current Drinking in Liver tissue. rs584007 is causal SNP with CLPP 0.1459

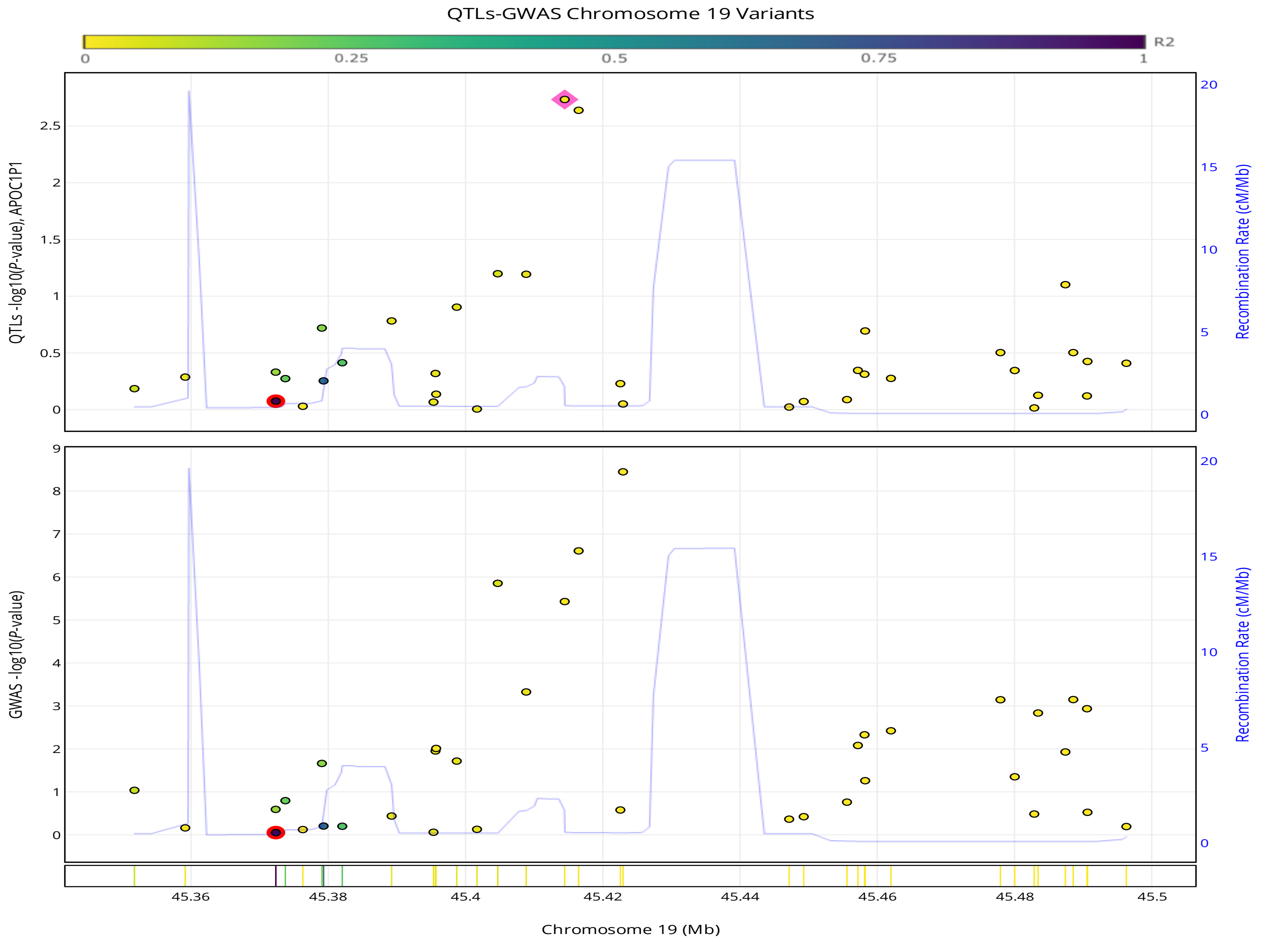

Colocalization between MT1DP gene and TG x Current Drinking in Liver tissue. rs584007 is causal SNP with CLPP 0.8305.

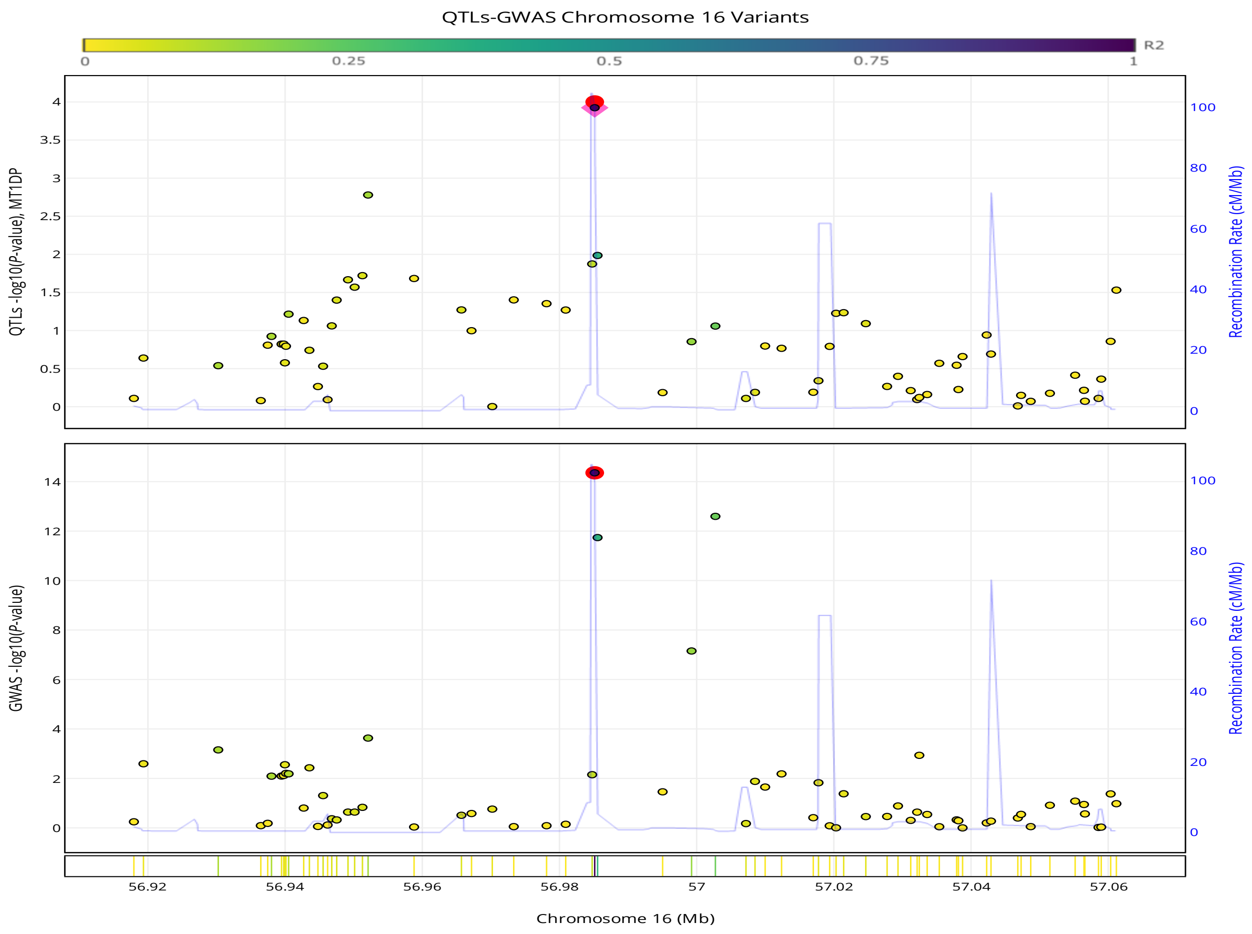

Colocalization between CETP gene and HDL-C x Ever Smoking in Stomach tissue. rs12720926 is a potential causal SNP with CLPP 0. 2736.

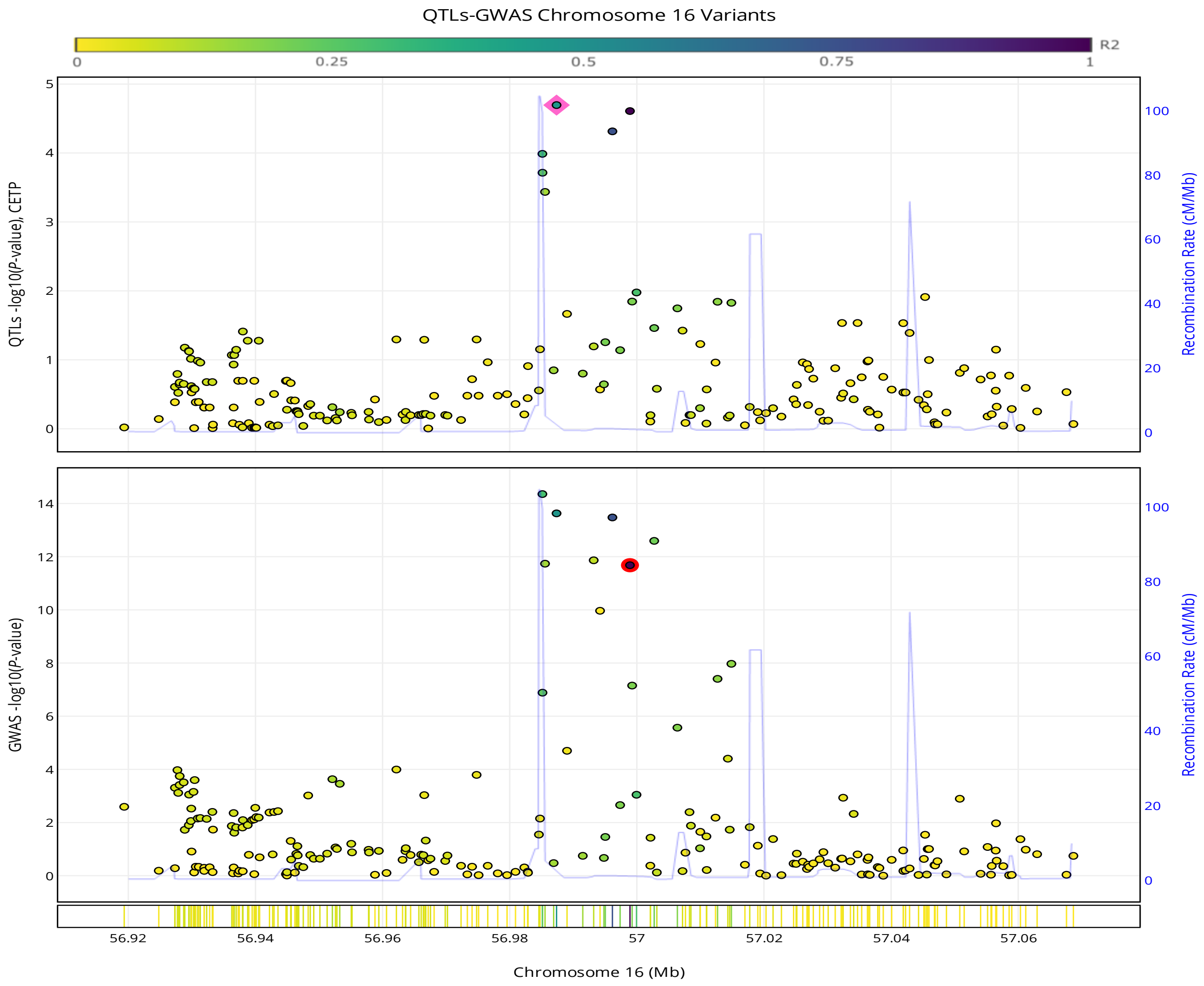

Colocalization between CETP gene and HDL-C x Ever Smoking in Stomach tissue. rs3816117 is a potential causal SNP with CLPP 0.1678.

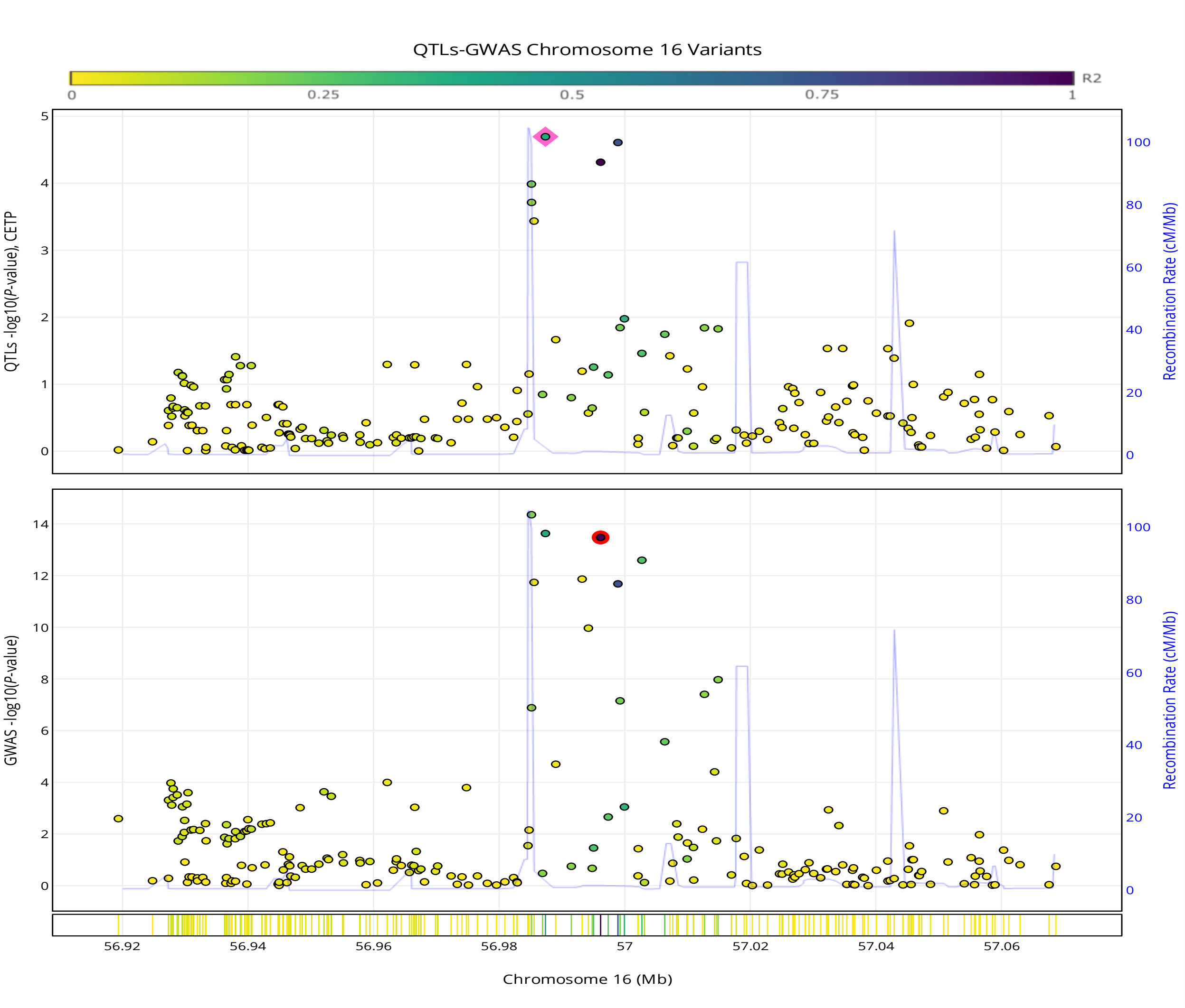

Supplementary Figure 15. The effect of adding a variant within the 500kb region of the SNP reported in Table 1. The significance (P-value of the interaction test) of the SNPs in table 1 have little changes, indicating no variants in the region can account for the interaction evidence.

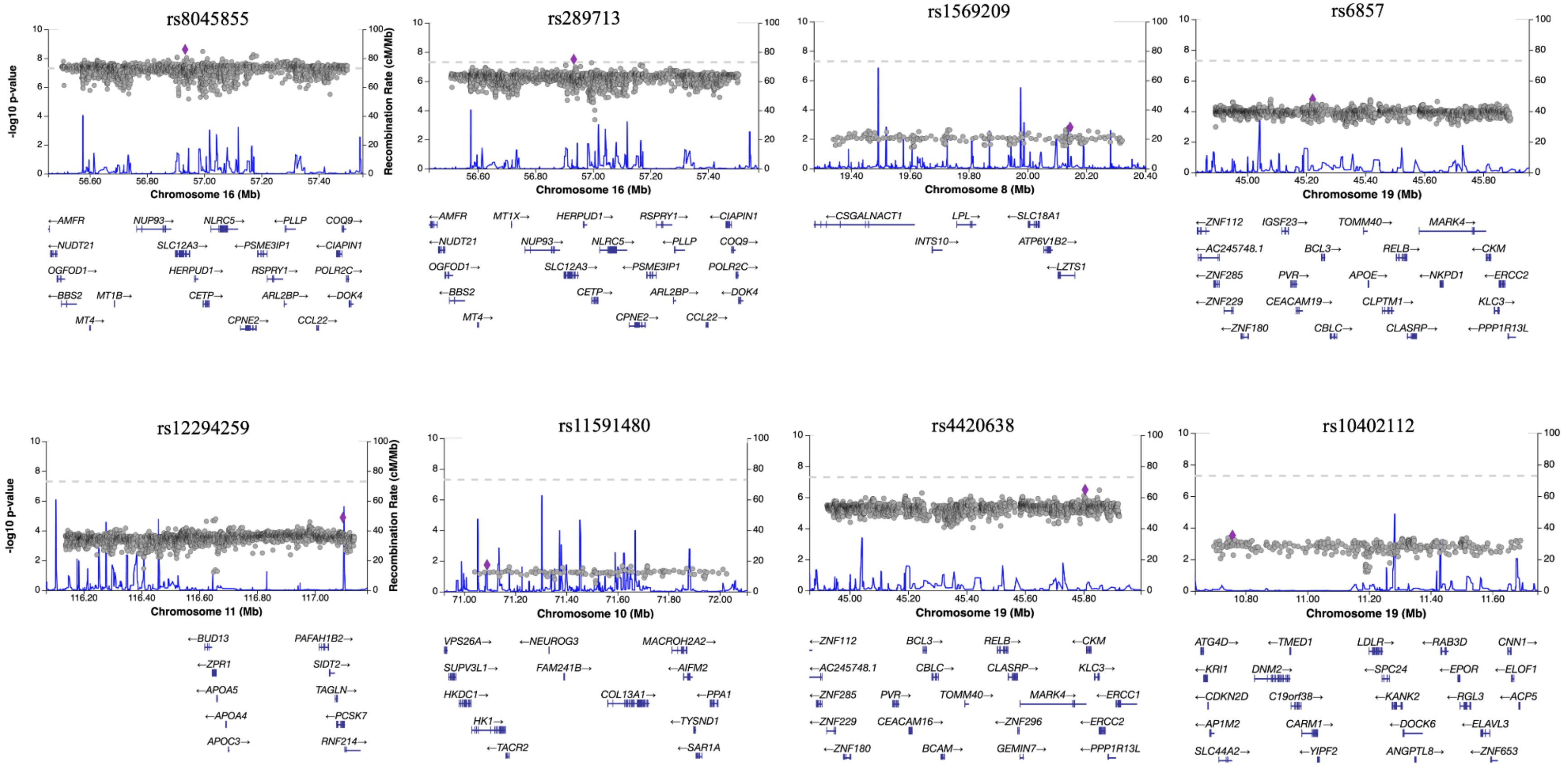

Regerences

1. H. Aschard, A perspective on interaction effects in genetic association studies. *Genet Epidemiol* **40**, 678-688 (2016).
