# Supplementary material for: An approach to identify gene-environment interactions and reveal new biological insight in complex traits
Source: Nat Commun. 2024 Apr 22;15:3385. doi: 10.1038/s41467-024-47806-3 (PMC11035594; doi:10.1038/s41467-024-47806-3)
Supplement: Supplementary file 3 — Description of Additional Supplementary Files [file 41467_2024_47806_MOESM3_ESM.pdf]

Description of Additional Supplementary Files

File Name: Supplementary Data 1
Description: Estimated θ and the corresponding SE by IMRP in cross-population and population specific analyses. Here θ is equivalent to the causal effect in Mendelian Randomization analysis when we consider GWIS and GWAS effect sizes as the exposure and outcome effect sizes, respectively. In the gene-environment interaction analysis, we analyzed cigarette smoking status (current, ever) and alcohol drinking (current, regular). (-) represents that GWIS summary statistics that are not available for TG and ever smoking in African ancestry population.

File Name: Supplementary Data 2
Description: Genomic control λ values in the genome wide analysis using T_{MR_GxE} in cross-population and population specific analyses. In the gene-environment interaction analysis, we analyzed cigarette smoking status (current, ever) and alcohol drinking (current, regular). (-) represents that GWIS summary statistics are not available for TG and ever smoking in African ancestry population.

File Name: Supplementary Data 3
Description: (a). Novel interaction loci detected by T_{MR_GxE} and the replication results by the direct G×E test in UK Biobank. At each locus, we reported the lead G×E SNP by T_{MR_GxE} test. EA: effect allele; GxL AF: effect allele frequency in the Gene-lifestyle interactions study; UKBB AF: effect allele frequency in UKBB data; GxL Main Effect: main effect in the Gene-lifestyle interactions study; GxL Main Effect SE: the standard error of the main effect in the Gene-lifestyle interactions study; UKBB Main Effect: main effect in the UKBB data; UKBB Main Effect SE: the standard error of main effect in UKBB; GLGC Marginal Effect: marginal effect in the GLGC study; GLGC Marginal Effect SE: the standard error of the marginal effect in the GLGC study; GxL Int Effect: interaction effect in the gene-lifestyle interactions study; GxE Int Effect SE: the standard error of the interaction effect size in the gene-lifestyle interactions study; UKBB Int Effect: the interaction effect in the UKBB data; UKBB Int Effect SE: the standard error of the interaction effect size in the UKBB data; GxL N: sample size in the gene-lifestyle interactions study; UKBB N: sample size of the UKBB data; PleioP_MR: p-value of T_{MR_GxE} test; GxL Int P: p-value of the direct test in the gene-lifestyle interactions study; UKBB Int P: p-value of the direct test in the UKBB data; GxL and UKBB P: p-value of the direct test in the meta-analysis of the gene-lifestyle interactions study and UKBB data; last column: Evidence of association with Smoking or alcohol drinking in Nature, Saunders et al 2022 (Y: yes; S:Smoking; A:alcohol drinking).
(b). Comparisons of the number of genome wide significant loci detected by the two-step method and the direct method in multiancestry analysis. Only variants with MAF>1% were counted. Two-step: T_{MR_GxE} test followed by the direct test. Direct test: the direct interaction test.

File Name: Supplementary Data 4
Description: Heritability estimates based on marginal effects and interaction/mediation effects in cross-population and European population. Marginal h2: the heritability estimated based on the marginal effect sizes in the GLGC study using LD score regression; Marginal h2 SE: the standard error of the marginal heritability estimate; Interaction/mediation h2: the heritability of GxE interaction or mediation estimated based on the effect sizes of the T_{MR_GxE} test; Interaction/mediation h2 SE: standard error of the estimated heritability of GxE interaction or mediation; (Interaction,mediation h2)/marginal h2: ratio of Interaction/mediation h2 over Marginal h2.
