# Supplementary material for: An approach to identify gene-environment interactions and reveal new biological insight in complex traits
Source: Nat Commun. 2024 Apr 22;15:3385. doi: 10.1038/s41467-024-47806-3 (PMC11035594; doi:10.1038/s41467-024-47806-3)
Supplement: Supplementary file 5 — Reporting Summary [file 41467_2024_47806_MOESM5_ESM.pdf]

nature portfolio

Corresponding author(s): Xiaofeng Zhu

Last updated by author(s): 3/11/2024

Reporting Summary

Nature Portfolio wishes to improve the reproducibility of the work that we publish. This form provides structure for consistency and transparency in reporting. For further information on Nature Portfolio policies, see our Editorial Policies and the Editorial Policy Checklist.

Statistics

For all statistical analyses, confirm that the following items are present in the figure legend, table legend, main text, or Methods section.

n/a	Confirmed	
☐	☒	The exact sample size (*n*) for each experimental group/condition, given as a discrete number and unit of measurement
☐	☒	A statement on whether measurements were taken from distinct samples or whether the same sample was measured repeatedly
☐	☒	The statistical test(s) used AND whether they are one- or two-sided<br>*Only common tests should be described solely by name; describe more complex techniques in the Methods section.*
☐	☒	A description of all covariates tested
☐	☒	A description of any assumptions or corrections, such as tests of normality and adjustment for multiple comparisons
☐	☒	A full description of the statistical parameters including central tendency (e.g. means) or other basic estimates (e.g. regression coefficient) AND variation (e.g. standard deviation) or associated estimates of uncertainty (e.g. confidence intervals)
☐	☒	For null hypothesis testing, the test statistic (e.g. *F*, *t*, *r*) with confidence intervals, effect sizes, degrees of freedom and *P* value noted<br>*Give P values as exact values whenever suitable.*
☒	☐	For Bayesian analysis, information on the choice of priors and Markov chain Monte Carlo settings
☐	☒	For hierarchical and complex designs, identification of the appropriate level for tests and full reporting of outcomes
☐	☒	Estimates of effect sizes (e.g. Cohen's *d*, Pearson's *r*), indicating how they were calculated

Our web collection on statistics for biologists contains articles on many of the points above.

Software and code

Policy information about availability of computer code

Data collection	We used the publicly available GWAS summary statistics and the UK Biobank data.<br>GLGC summary statistics, http://csg.sph.umich.edu/willer/public/glgc-lipids2021,<br>CHARGE GLI summary statustics, https://www.ncbi.nlm.nih.gov/projects/gap/ study ID: phs000930.v10.p1<br>UK Biobank, https://biobank.ctsu.ox.ac.uk.<br>1000G reference, https://privefl.github.io/bigsnpr/reference/download_1000G.html<br>GTEx, http://www.gtexportal.org<br>ezQTL (https://analysistools.cancer.gov/ezqtl/#/home
Data analysis	T_(MR_GxE) test was used the software IMRP which is available in the Github repository with the following link, https://github.com/XiaofengZhuCase/IMRP. Heritability analysis was performed by Bigsnpr, https://privefl.github.io/bigsnpr/reference/snp_ld_scores.html and LDSC regression, https://github.com/bulik/ldsc. FUMA: https://fuma.ctglab.nl/. Software ezQTL: https://analysistools.cancer.gov/ezqtl/#/home. MAGMA: https://ctg.cncr.nl/software/magma. DEPICT: https://github.com/perslab/depict. Plink: https://www.cog-genomics.org/plink2/. The codes for performing simulations and G×E for lipids were deposited in the Zenodo database:https://zenodo.org/records/10815731

For manuscripts utilizing custom algorithms or software that are central to the research but not yet described in published literature, software must be made available to editors and reviewers. We strongly encourage code deposition in a community repository (e.g. GitHub). See the Nature Portfolio guidelines for submitting code & software for further information.

Data

Policy information about availability of data

All manuscripts must include a data availability statement. This statement should provide the following information, where applicable:
- Accession codes, unique identifiers, or web links for publicly available datasets
- A description of any restrictions on data availability
- For clinical datasets or third party data, please ensure that the statement adheres to our policy

All the data are applicably available. The marginal summary statistics of high-density lipoprotein cholesterol (HDL-C), low-density lipoprotein cholesterol (LDL-C), and triglycerides (TG) from the Global Lipids Genetics Consortium study (GLGC, n=1.65M) 3 were downloaded at http://csg.sph.umich.edu/willer/public/glgc-lipids2021. GLGC consists of GWAS results from 1.65M subjects representing five genetic ancestry groups: European (N = 1.32M); African or admixed African (N = 99K); East Asian (N = 146K); Hispanic (N = 48K); and South Asian (N = 41K). We did not perform South Asian specific analysis because there was no corresponding GWIS in the CHARGE consortium. The GWIS summary statistics from CHARGE GLI working group in this study are available via dbGaP (accession number phs000930).

Research involving human participants, their data, or biological material

Policy information about studies with human participants or human data. See also policy information about sex, gender (identity/presentation), and sexual orientation and race, ethnicity and racism.

Reporting on sex and gender	We did not perform sex specific analysis. The summary statistics and UK Biobank data include both males and females.
Reporting on race, ethnicity, or other socially relevant groupings	We included European, African, South Asian, East Asian and Hispanic populations.
Population characteristics	We included European, African, South Asian, East Asian and Hispanic populations.
Recruitment	This study use existing data.
Ethics oversight	This study is approved by the IRB office at Case Western Reserve University, with the IRB number: STUDY20180592.

Note that full information on the approval of the study protocol must also be provided in the manuscript.

Field-specific reporting

Please select the one below that is the best fit for your research. If you are not sure, read the appropriate sections before making your selection.

☒ Life sciences ☐ Behavioural & social sciences ☐ Ecological, evolutionary & environmental sciences

For a reference copy of the document with all sections, see nature.com/documents/nr-reporting-summary-flat.pdf

Life sciences study design

All studies must disclose on these points even when the disclosure is negative.

Sample size	The marginal summary statistics of HDL-C, LDL-C, and TG from the Global Lipids Genetics Consortium study (GLGC, n=1.65M) were downloaded at http://csg.sph.umich.edu/willer/public/glgc-lipids2021.<br>GLGC consists of GWAS results from 1.65M subjects representing five genetic ancestry groups: European (N=1.32M); African or admixed African (N=99k); East Asian (N=146k); Hispanic (N=48k); and South Asian (N=41k). UK Biobank data has a sample size N=445,424.
Data exclusions	We did not exclude data.
Replication	We used the UK Biobank data to replicate the findings of GxE interactions.
Randomization	This is not a randomized experiment.
Blinding	Not applicable.

Reporting for specific materials, systems and methods

We require information from authors about some types of materials, experimental systems and methods used in many studies. Here, indicate whether each material, system or method listed is relevant to your study. If you are not sure if a list item applies to your research, read the appropriate section before selecting a response.

Materials & experimental systems

n/a	Involved in the study
☒	☐ Antibodies
☒	☐ Eukaryotic cell lines
☒	☐ Palaeontology and archaeology
☒	☐ Animals and other organisms
☒	☐ Clinical data
☒	☐ Dual use research of concern
☒	☐ Plants

Methods

n/a	Involved in the study
☒	☐ ChIP-seq
☒	☐ Flow cytometry
☒	☐ MRI-based neuroimaging
